# System-level synchronization and subtype-specific modules govern brain-wide serotonin axon innervation

**DOI:** 10.1073/pnas.2611533123

**Published:** 2026-09-22

**Authors:** Annabel M. J. Adams, Kenneth Lap Kei Wu, Zhongyu Li, Peiqi Li, Hangzhen Pan, Zuqi Huang, Sara Migliarini, Richard Dear, Bo Sun, Zora Chui-Kuen Chan, Jingwen Liang, Hongjie Li, Petra E. Vértes, Massimo Pasqualetti, Alina Isakova, Jing Ren

**Affiliations:** ^a^Neurobiology Division, The Medical Research Council Laboratory of Molecular Biology, Cambridge CB2 0QH, United Kingdom; ^b^ Qingyuan Research Institute, School of Computer Science, Shanghai Jiao Tong University, Shanghai 200240, China; ^c^ Department of Intelligent Systems, School of Software Engineering, Xi’an Jiaotong University, Xi’an 710049, China; ^d^ Department of Biology, Unit of Cell and Developmental Biology, University of Pisa, Pisa 56127, Italy; ^e^ Department of Psychiatry, University of Cambridge, Cambridge CB2 0SZ, United Kingdom; ^f^ Huffington Center on Aging, Baylor College of Medicine, Houston, TX 77030; ^g^ Department of Molecular and Human Genetics, Baylor College of Medicine, Houston, TX 77030; ^h^ Brain Resilience Lab, Knight Initiative for Brain Resilience, Wu Tsai Neuroscience Institute, Stanford University, Stanford, CA 94305

**Keywords:** serotonin, development, transcriptomics, wiring specificity, whole brain mapping

## Abstract

The serotonin system modulates nearly all neural circuits across development and adulthood and is a major pharmacological target for mood and anxiety disorders. Forebrain-projecting serotonin neurons are organized into projection-defined subsystems that align with molecular heterogeneity, but how these subsystems assemble during development remains unclear. We combined whole-brain developmental projection maps with single-cell transcriptomics to define the spatiotemporal and molecular logic of postnatal serotonin innervation. Using a deep-learning pipeline, we generated four-dimensional whole-brain maps of postnatal serotonin axon density and uncovered two coupled modes of regulation: region-specific trajectories of axon growth and refinement, and an early-postnatal, brain-wide synchronization of innervation. To link these dynamics to cell-intrinsic programs, we profiled single-cell transcriptomes of forebrain-projecting serotonin neurons from embryonic through postnatal stages and reconstructed developmental trajectories. We found that serotonin neuron heterogeneity was established embryonically and that the subtype taxonomy was largely conserved between mice and humans. Across serotonin subtypes, we observed a coordinated surge in wiring-molecule expression at mouse postnatal day 4 that aligned with the subsequent global innervation wave. Tracing subtypes defined by developmentally conserved markers at birth revealed complementary projection territories and engagement of distinct postnatal wiring modules that matched region-specific terminal maturation. Together, these results show that brain-wide serotonin innervation is built through embryonic subtype specification and two layers of postnatal control: system-level synchronization and subtype-specific terminal development.

## Significance

Serotonin shapes nearly every brain circuit and is a major target of psychiatric drugs, yet how its widespread pathways are assembled has remained unclear. We combined whole-brain developmental mapping with single-cell RNA profiling to show that forebrain-projecting serotonin neurons are specified into subtypes before birth and then wire the brain through two coordinated postnatal programs: an early, brain-wide synchronization of axon growth and region-specific terminal maturation. This framework replaces the traditional nonspecific “space-filling” view with a genetically guided, staged developmental plan that is conserved across species. Our four-dimensional atlas and molecular timelines identify critical developmental windows and mechanisms that can inform models of circuit function, vulnerability, and therapeutic timing.

The serotonin system is the primary target of pharmacological treatments for mental disorders, such as depression and anxiety, which are among the leading causes of global disability (1, 2). It plays a crucial role in modulating diverse brain functions and shaping neural development (3).

Although serotonin neurons constitute only ~1/200,000 of all neurons in the human brain, they exert a broad influence over nearly all brain circuits through extensive axonal projections. In particular, serotonin neurons located in the dorsal raphe (DR) and median raphe (MR) nuclei innervate the entire forebrain and are implicated in regulating various mental states and behaviors, including mood, impulsivity, aggression, fear, learning, reward, social interaction, etc (4). In addition to their roles in adulthood, serotonin neurons are also involved in modulating synapse formation and shaping forebrain network architecture during development (3, 5). Historically, the DR serotonin neurons have been viewed as a relatively monolithic population, modulating downstream brain regions via diffuse volume transmission, with little synaptic or regional specificity. This led to the longstanding hypothesis that serotonin projections follow a tiled, space-filling innervation strategy. However, our previous work has overturned this notion, demonstrating that the DR serotonin system is in fact heterogeneous and comprises projection-defined subsystems with distinct input–output connectivity, physiological properties, and behavioral functions (6). Subsequent transcriptomic profiling of DR and MR serotonin neurons further revealed the heterogeneity at the molecular level (7, 8).

Dysregulated serotonin transmission during development has been linked to emergence of adulthood behaviors related to mental disorders in rodent models (9). Disrupted differentiation of raphe serotonin precursors results in heightened anxiety and aggressive behavior in adulthood (10, 11). Likewise, perturbations in serotonin receptor signaling during early postnatal periods produce persistent anxiety- and depression-like phenotypes that cannot be reversed by gene reactivation in adulthood (12). Furthermore, blocking the serotonin transporter (*Sert*) at early to mid-postnatal days alters emotional behavior in adult mice (13).

Despite their diverse and fundamental roles in neural development and behavior, it remains unclear when heterogeneity arises among forebrain-projecting serotonergic neurons during development and how these cells establish extensive yet target-specific innervation. Here, we leverage recent advances in machine-learning–assisted 3D whole-brain mapping and single-cell transcriptomics to construct a comprehensive, multimodal developmental trajectory of the forebrain-projecting serotonin system. We found that serotonin neuron heterogeneity is embryonically established and evolutionarily conserved. We identified key windows of circuit assembly and showed that, although serotonergic signaling largely relies on volume transmission, the development of forebrain projections follows a highly specific, genetically encoded program coordinated at the system level.

## Results

### Spatiotemporal Mapping of Serotonin Axon Innervation across the Whole Brain Reveals Dual Scale Regulation.

Earlier studies have shown that serotonin axons reach all major brain targets by P0 (14), but the brain-wide spatiotemporal dynamics of their subsequent postnatal innervation remain poorly defined.

**Fig. 1.**
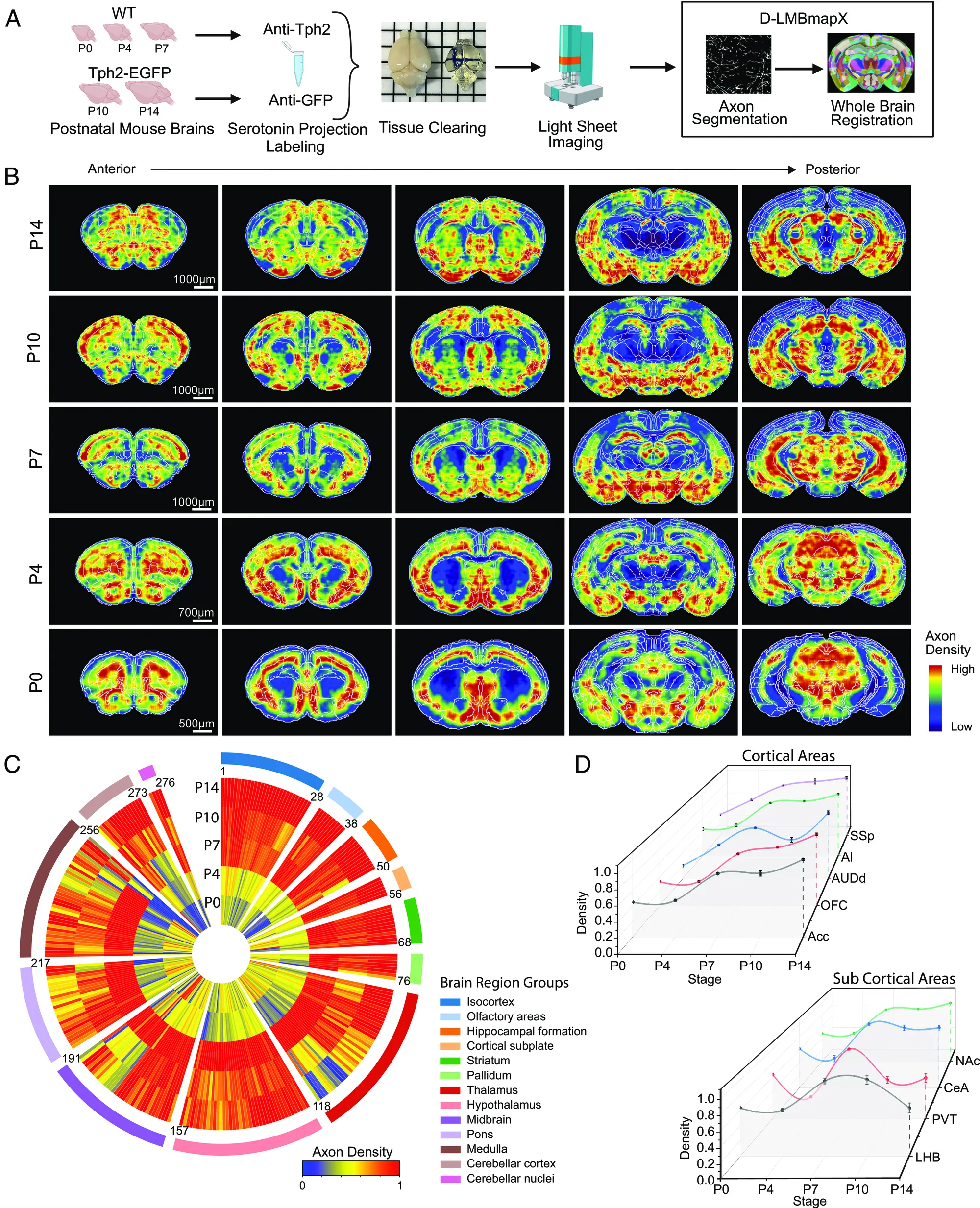
Spatiotemporal mapping of serotonin axon innervation across the whole brain. (*A*) Experimental and analytical workflow for whole brain profiling of serotonin axon projections across five postnatal stages. Serotonin axons in wild-type mouse brains from P0, P4, and P7 were labeled by anti-Tph2 staining. Serotonin axons in *Tph2-eGFP* mouse brains from P10 and P14 were labeled by anti-GFP staining. The brains subsequently underwent tissue-clearing and light-sheet imaging. D-LMBmapX was used for image analysis and atlas construction. (*B*) Coronal heatmaps of whole brain serotonin axonal innervation patterns at five positions along the anterior–posterior axis for five postnatal stages (n = 3 brains for each stage). The color gradient denotes scaled axon density across the whole brain. (*C*) Radial plot illustrating the temporal progression in serotonin axon density across all 276 profiled brain regions. Individual brain regions are grouped according to their broader anatomical categories (Dataset S1). Color code indicates scaled axonal density across developmental stages for each brain region. (*D*) Ridge plots showing the axon density changes across developmental stages for highlighted cortical (*Top*) and subcortical (*Bottom*) brain regions. For the shown brain regions, the following axon density changes between timepoints are significant (*P* < 0.05); P7–P0, P10–P0, P14–P0 for Acc, OFC, and AI; P4–P0, P7–P0, P10–P0, P14–P0 for AUDd and SSP; P10–P0 for LHb; P4–P0, P7–P0 for PVT, P7–P0, P10–P0, P14–P0 for CeA and NAc (n = 3 brains).

To systematically investigate when and how serotonergic neurons establish their local innervation of the target regions, we performed 3D brain-wide profiling of serotonin axonal projections across five postnatal (P) stages (P0, P4, P7, P10, and P14, n = 3 mice). Serotonin axons were labeled and visualized using tissue clearing and light-sheet imaging. Automated and precise axon segmentation, along with whole-brain registration, was accomplished using D-LMBmapX (15), our newly established pipeline tailored specifically for developmental mouse brains (Fig. 1*A* and Movie S1). To generate a spatiotemporal atlas of serotonin axon innervation, we imaged mouse brains for the five stages (Fig. 1*B*) and quantified serotonin axon densities across more than 260 individual brain regions (Fig. 1*C* and Movie S2). Of the 13 major brain areas analyzed, the cortical areas, hippocampal formation, striatum, pallidum, and cerebellum broadly exhibited a monotonic increase in serotonin axonal innervation. In contrast, the hypothalamus plateaued by P7 and the thalamus, pons, and medulla showed a significant but transient increase in serotonin axon density during postnatal development (Fig. 1*C* and Dataset S1).

Within each major brain area, we observed that serotonin axonal innervation followed a highly heterogeneous and region-specific pattern rather than progressing uniformly. These include distinct trajectories such as, sharp increase in axon density at specific time points, transient peaks, stable levels from birth, or even a general decrease over time. Notably, these temporal patterns did not necessarily correlate with anatomical proximity. For example, the LHb and the paraventricular nucleus of the thalamus (PVT) are adjacent structures, but they appeared independent innervation pattern (Fig. 1*D*).

Despite these spatiotemporal dynamics, quantitative analysis across all profiled brain regions revealed a pronounced and synchronized surge in axon density occurred between P4 and P7, particularly across forebrain targets (Fig. 1*C*), indicating a critical time window for terminal field development.

Together, these findings support a model in which serotonin axon innervation is governed by coordinated spatial and temporal programs at two scales: region-specific regulation that imposes distinct local timing, and a system-level mechanism that orchestrates a brain-wide wave of innervation.

### Postnatal Wiring Program Encodes System-Level Coordinated Serotonin Innervation.

Next, we asked how the dual-scale organization of serotonin axon innervation is established during development.

**Fig. 2.**
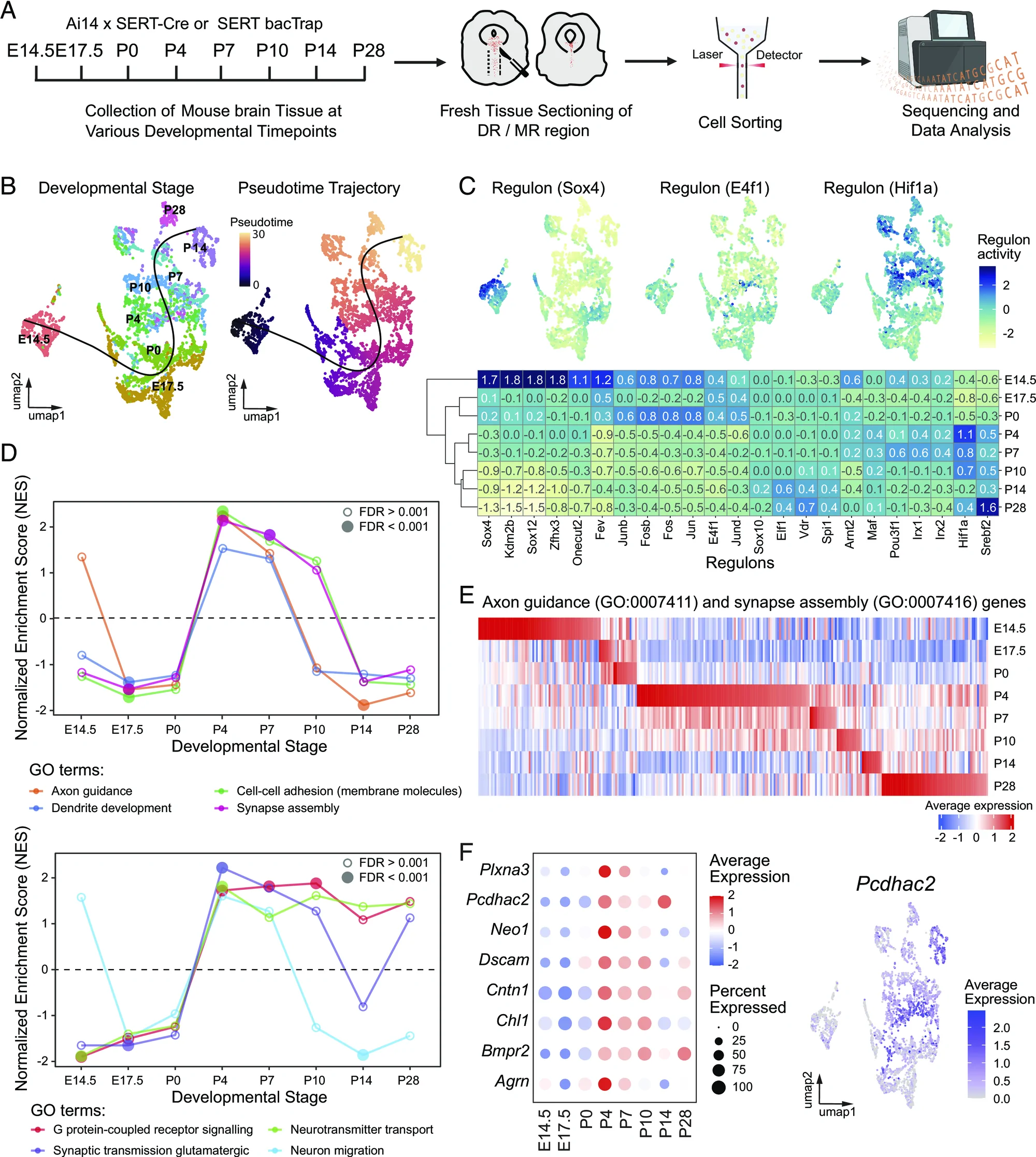
Transcriptomic programs for serotonin innervation. (*A*) Experimental workflow for generating a scRNA-seq dataset from eight developmental stages. DR & MR regions of mice at eight developmental stages, genetically labeled serotonin neurons were isolated by FACS for scRNA-seq using 10× Genomics pipeline. (*B*) Single cell transcriptomes were visualized in a UMAP (*Left*) and color-coded by developmental stage, and a pseudotime trajectory was fitted across eight developmental stages (*Right*). (*C*) *Top*, heatmap showing z-score of representative regulons active in embryonic, early postnatal and late postnatal stages. Bottom, heatmap of z-scores showing stage-specific activity of regulons predicted by pySCENIC. (*D*) GO terms related to axon growth, synapse formation, and neuronal function were significantly enriched at P4, preceding the onset of the brain-wide wave of serotonin innervation. Notably, these gene sets were downregulated at later stages while enrichment of those for G-protein–coupled signaling and neurotransmitter transport persisted. Filled circles indicate FDR < 0.001. (*E*) Heatmap showing developmental stage-dependent profile of expression of axon guidance and synapse assembly genes sorted by expression peaking time. Only genes that were abundantly expressed by more than 50% of serotonin neurons in at least one stage are shown. 96 out of 283 (34%) abundantly expressed axon guidance and synapse assembly genes had peak expression at P4. (*F*) *Left*, dotplot showing expression of representative axon guidance genes in developmental scRNA^DR&MR^ datasets. *Right*, tSNE plot showing expression of the axon guidance gene *Pcdhac2* in single cells.

In mice, serotonin neurons are generated around embryonic day (E) 11, and their axons initiate the long-range growth along major brain tracts starting at E13.5 (16, 17). We systematically profiled the transcriptomes of serotonin neurons from the DR and MR across eight developmental stages (E14.5, E17.5, P0, P4, P7, P10, P14, and P28). To isolate serotonin neurons, we crossed a Cre reporter mouse line Ai14 (18) with *Sert-Cre* mice (19) to drive cell type–specific fluorescent protein expression and performed fluorescence-activated cell sorting (FACS). Sorted neurons were then analyzed using single-cell RNA sequencing (scRNA-seq) with 10× Genomics platform (Fig. 2*A*).

In total, we collected 8,189 single-cell transcriptomic profiles (*SI Appendix*, Fig. S1*A*). Serotonergic markers and associated lineage genes, including *Slc6a4 (Sert)*, *Tph2*, *Lmx1b*, *Fev (Pet1)*, *Slc18a2 (Vmat2)*, *Maoa*, *Maob*, and *Ddc*, were detected in nearly all profiles, confirming stable serotonergic identity across development. Among these genes, Vmat2 and Maob showed increased abundance during postnatal maturation (*SI Appendix*, Fig. S1*B*). Uniform manifold projections (UMAP) of the full dataset arranged serotonin neurons from different time points along a continuous developmental trajectory, in agreement with pseudotime inferred by Slingshot (20) (Fig. 2*B* and *SI Appendix*, Table S1). We then examined which gene regulatory networks drove the developmental trajectory of serotonin neurons by using pySCENIC (21) for regulon activity detection. Several regulons displayed specific temporal activity, such as Sox4, Kdm2b, Sox12, and Zfhx3, which were found to highly expressed at E14.5, Hif1a at early-mid postnatal stages, and Srebf2 at late postnatal stages (Fig. 2*C*). Regarding neonatal stages associated regulons, Fev (22) showed strong activity at E14.5 and gradually decreased as development progresses, and E4f1 appeared consistent activity. Interestingly, several immediate-early genes from the AP-1 family showed strong activity at P0, including *Fos*, *Fosb*, *Jun*, and *Junb* (Fig. 2*C*). Additionally, we identified stage-specific marker genes, highlighting unique molecular signatures at various points in development (*SI Appendix*, Fig. S1*C*).

To assess the temporal expression pattern of genes that may instruct the global serotonin axon innervation, we performed stage-wise Gene Set Enrichment Analysis (GSEA) of Gene Ontology (GO) Biological Process terms related to neuronal development and summarized normalized enrichment (NES). At the whole population level, enrichment of wiring-related gene sets such as axon guidance and synapse assembly rose sharply at P4, with sustained elevation at P7, and decreased after P10 (Fig. 2*D*). One third of axon guidance and synapse assembly genes that were expressed in a majority of serotonin neurons had peak expression at P4 (Fig. 2*E*). We also found that Pcdhac2, a known regulator of serotonin axon terminal density (23), was broadly expressed across serotonin neurons and became elevated after P4 (Fig. 2*F*). Notably, the peak activation of wiring-related transcriptional programs occurred at P4, preceding the brain-wide surge in serotonin axon density observed between P4 and P7, consistent with a coordinated developmental program that supports subsequent terminal field expansion.

### Serotonin Neurons Commit to Adulthood Heterogeneity during Embryogenesis.

Previously, we characterized the molecular architecture of the DR and MR serotonin neurons by scRNA-seq with Smart-seq2 platform in adult mouse brains (referred to as the “Smart-seq2 adult^DR&MR^ dataset”) (7). This analysis identified six molecularly distinct clusters in the principal DR (DR), four clusters in the MR, and one caudal DR (cDR) cluster. Here, we aimed to determine when this heterogeneity begins to emerge and whether the transcriptomic commitment of subtype identities is behind the dual-scale organization of serotonin axon innervation.

**Fig. 3.**
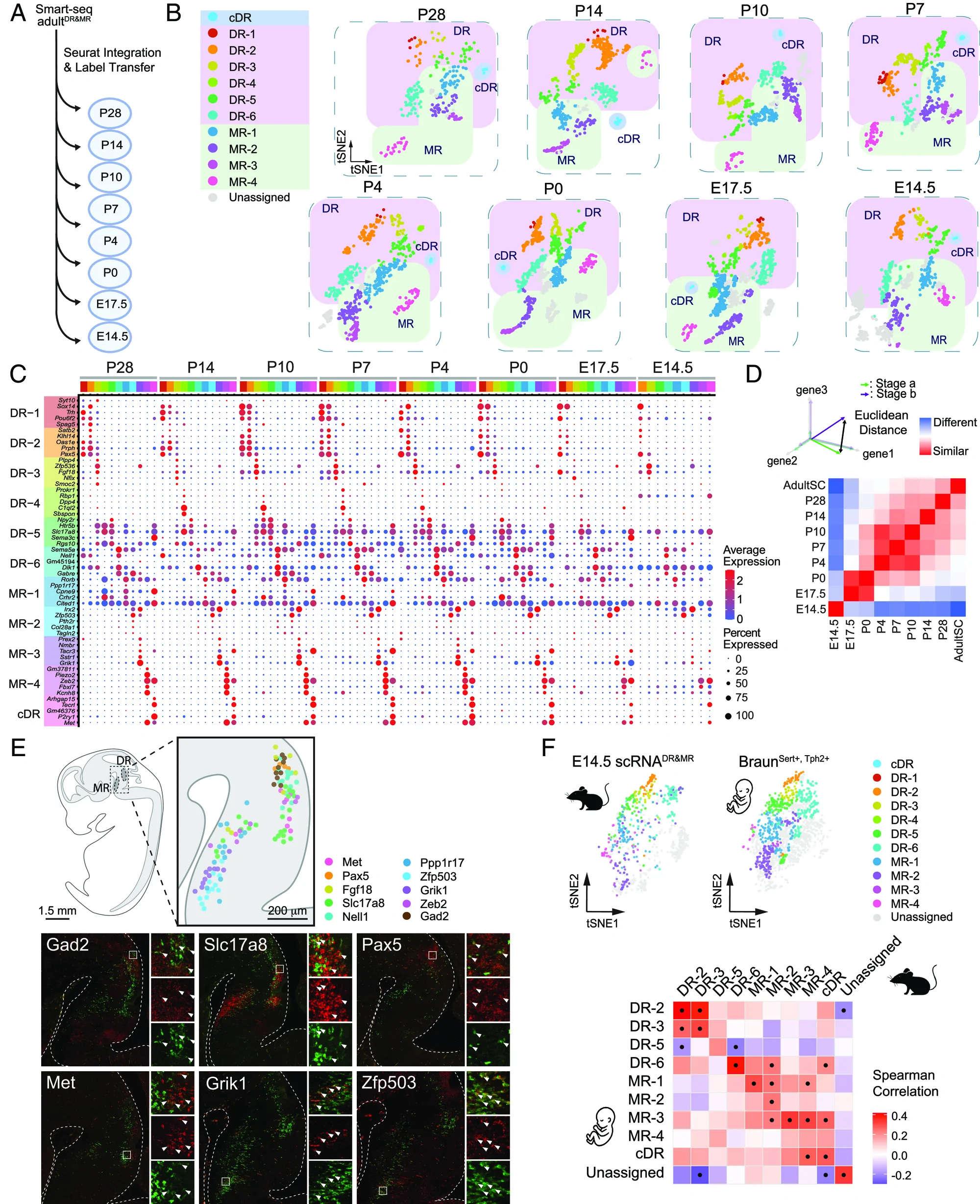
Serotonin neuron subtypes are determined in early developmental stages. (*A*) Analytical workflow for transfer of natural clusters to scRNA^DR&MR^ datasets by integration with Smart-seq2 adult^DR&MR^ dataset and knn-based label transfer. (*B*) tSNE plots of single-cell transcriptomic profiles of the serotonin neuron subtypes in scRNA^DR&MR^ datasets. Cells are colored according to subtype labels transferred from Smart-seq2 adult^DR&MR^ dataset after integration. (*C*) Expression patterns of marker genes, selected from distinct clusters in the Smart-seq2 adult^DR&MR^ dataset, across single-cell transcriptomic profiles spanning eight developmental stages. (*D*) Heatmap showing Euclidean distances between single-cell transcriptomic profiles of serotonin neurons across embryonic, postnatal, and adult stages. Distances are calculated based on the expression of the top 10 marker genes from different clusters. (*E*) *Top* panel: Schematic showing spatially segregated expression of subtype markers selected from (*C*) in embryonic day 14.5 embryo. *Bottom* panels: Representative images for Gad2, Slc17a8, Pax5, Met, Grik1 and Zfp503 are shown. Green cells are Sert positive, with mRNA of the indicated marker gene in red. *Inset* panels show a magnified view of the boxed area. White arrows point to Sert positive cells expressing the marker gene. Images for remaining genes shown in the schematic diagram are shown in *SI* *Appendix*, Fig. S4. (*F*) tSNE plots showing Sert and Tph2 double-positive cells in human embryonic scRNA dataset and serotonin neurons in E14.5 scRNA^DR&MR^ dataset. Cells in the human data are colored according to subtype labels transferred from scRNA^DR&MR^ dataset after integration. Interspecies Spearman correlation was calculated based on common highly variable genes. Dots indicate FDR < 0.05.

To test whether the transcriptomic subtypes observed in mature serotonin neurons can be traced back through development, we computationally mapped developmental cells onto adult projection-defined subtypes. We first integrated each developmental transcriptomic dataset with the annotated Smart-seq2 adult^DR&MR^ reference by Seurat CCA (Fig. 3*A*; see *Materials and Methods*). This approach revealed a striking correspondence between transcriptional identities at each developmental stage and adulthood subtypes, which held true across all eight developmental stages—even as early as E14.5 (Fig. 3*B* and *SI Appendix*, Table S2). To assess whether this result depended on the specific integration strategy, we quantified stage- and subtype-specific label transfer confidence and the fraction of unassigned cells, and repeated the analysis using alternative integration approaches. Integration methods based on distinct computational frameworks, including RPCA, JPCA, and scVI, recovered broadly similar developmental-to-adult subtype correspondences. Although the fraction of cells assigned to adult reference subtypes varied across methods, concordant assignments were highly consistent, supporting an underlying relationship between developmental and adult serotonergic subtype identities that is reproducible across integration frameworks (*SI Appendix*, Fig. S2). We further asked whether a similar structure was present in the developmental data without adult-reference anchoring. Natural clustering of the developmental datasets closely recapitulated the subtype structure inferred by CCA-based label transfer, as assessed by both Jaccard index and Spearman correlation. This agreement was already evident at E14.5, indicating that the developmental subtype structure is not imposed by reference-based label transfer and is already present within the embryonic dataset itself (*SI Appendix*, Fig. S3).

The cell distributions in the DR, MR, and cDR clusters across all tSNE plots of the developmental datasets exhibited clear segregation (Fig. 3*B* and *SI Appendix*, Fig. S3*B*). To test whether transferred subtype labels were consistent with anatomical identity, we dissected the DR from P7 brain sections and performed scRNA-seq on serotonin neurons from this DR-specific dataset (DR-only P7) (*SI Appendix*, Fig. S3*A*). After integration with the adult reference and label transfer, the great majority of cells mapped to DR subtypes (*SI Appendix*, Fig. S3 *B* and *C*), consistent with their anatomical origin. A small subset of cells was assigned to the MR-1 subtype, likely reflecting the difficulty of achieving precise anatomical dissection (7, 24). By fitting a pseudotime curve to the developmental datasets with Slingshot (20), we identified distinct developmental trajectories for DR and MR serotonin neurons. At E14.5, DR serotonin neurons occupied an earlier position on the pseudotime scale compared to MR neurons, but prenatally, DR serotonin neurons exhibited a steeper trajectory of maturation than MR neurons. Postnatally, DR neuron development slowed, and DR neurons did not reach a mature state comparable to MR neurons until around P14, resulting in a significant difference in their overall pseudotime profiles (*SI Appendix*, Fig. S3*D*).

To track the emergence of molecularly defined serotonin neuron subtypes during development, we examined the expression of genes that distinguish adult serotonergic subtypes across developmental stages. Notably, key molecular characteristics that segregate mature serotonin neuron subtypes were already present among embryonic serotonin neurons, revealing an organization consistent with the adult subtype taxonomy (Fig. 3*D*). The segregation sharpened during early postnatal maturation, further illustrated by Euclidean distance measurements (Fig. 3*E*). Quantitatively, Euclidean distances between subtype marker-expression profiles across stages revealed two epochs of accelerated divergence, E14.5 to E17.5 and P0 to P4, indicating discrete transitions in subtype maturation rather than gradual drift (Fig. 3*E*). To further validate the emergence of serotonergic subtype organization during embryonic development, we examined the spatial expression of marker genes that distinguish adult serotonergic subtypes and retain subtype-enriched expression at E14.5. Based on our previous characterization of adult serotonin neuron subtypes (7), these populations occupy distinct anatomical territories within the DR and MR. We therefore asked whether markers associated with specific adult subtypes already exhibit corresponding spatial organization at E14.5. Fluorescent in situ hybridization (FISH) for marker gene mRNAs revealed spatially segregated expression patterns that recapitulated the broad anatomical arrangement of the corresponding adult subtype groups (Fig. 3*E* and *SI Appendix*, Fig. S4). These observations provide independent anatomical support for the subtype identities inferred by CCA label transfer and indicate that the overall spatial framework of serotonergic subtype organization is already established by E14.5 (Fig. 3*E*). These results indicate that the adulthood heterogeneity of serotonin neurons is already evident at embryonic stages. Consistent with this early specification, serotonin receptors, and associated signaling pathways, together with selected neuropeptides, neuropeptide receptors, and cholinergic and monoaminergic receptors that show subtype-specific expression in adulthood, were already detectable at embryonic stages and largely maintained their subtype-specific patterns into adulthood (7) (*SI Appendix*, Figs. S5 and S6). These findings suggest that functional features of serotonergic subtype identity emerge alongside the early molecular divergence of serotonin neurons.

To determine whether the molecular diversity of serotonin neurons observed in mice is conserved in humans, we aligned our mouse E14.5 dataset with a published scRNA-seq dataset of human prenatal brain cells (25). Interestingly, we found that the taxonomy of subtypes is largely conserved between humans and mice (Fig. 3*F*).

### Projection-Specific Subtypes Employ Translationally Distinct Wiring Regulators.

Because most serotonergic axons have not yet reached their forebrain targets by E14.5, we wonder whether these embryonically emerged subtypes correspond to future projection targets.

**Fig. 4.**
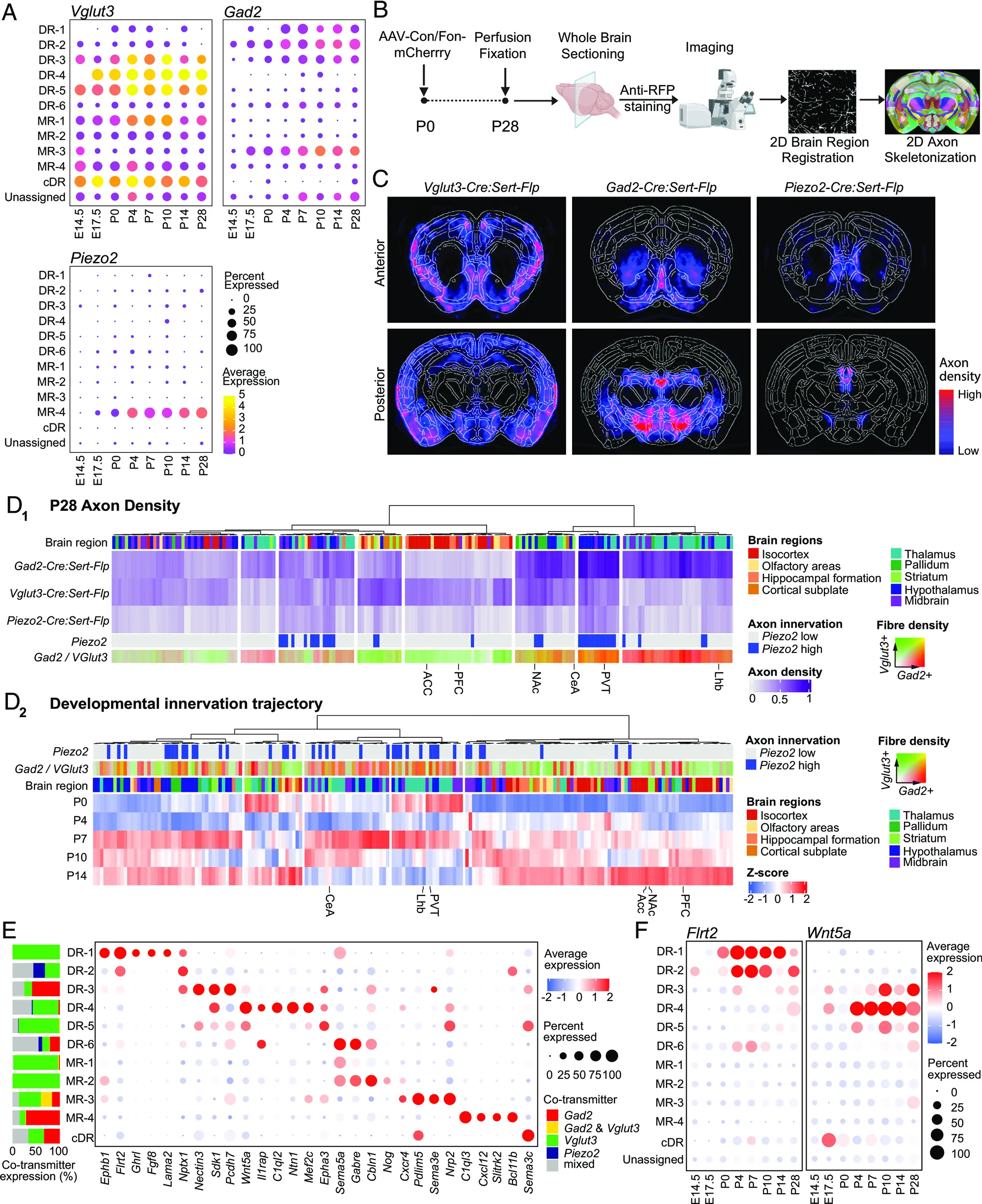
Functional subtypes determine target location while genetic subtypes determine innervation timeline. (*A*) Expression patterns of marker genes—*Vglut3*, *Gad2*, and *Piezo2*—showing temporal and serotonin neuron subtype specificity across development. (*B*) Schematic showing experimental design for intersectional labeling at P0 and automated axon density quantification at P28 using D-LMBmapX for automated axon segmentation and brain region registration. (*C*) Representative coronal heatmaps showing the regional axon density of Vglut3^+^, Gad2^+^, and Piezo2^+^ serotonin neurons at P28, respectively. (*D*_1_) Heatmap of midbrain and forebrain densities of *Vglut3*^+^, *Gad2*^+^, and *Piezo2*^+^ serotonin neurons at P28. Axon innervation labels *Gad2/Vglut3* show the relative density of *Vglut3*^+^ and *Gad2*^+^ axons in each brain region as a gradient. Regions with *Piezo2*^+^ serotonin neuron density z-score > 1 are labeled as *Piezo2* high. Axon densities of *Vglut3*^+^, *Gad2*^+^, and *Piezo2*^+^ serotonin neurons in hindbrain and cerebellum at P28 are shown in *SI Appendix*, Fig. S3_1_. (*D*_2_) Heatmap of the developmental profile for serotonin neuron innervation in midbrain and forebrain regions. Axon densities are normalized per brain region. Axon innervation labels for each brain region are inherited from D_1._ Innervation profiles for hindbrain and cerebellum are shown in *SI Appendix*, Fig. S3*A*_2_. (*E*) Heatmap showing differential expression of axon guidance and synapse assembly genes by serotonin subtypes at P4. The bar chart on the left shows the composition of serotonin neurons expressing *Vglut3*^+^, *Gad2*^+^, *Vglut3*^+^ & *Gad2*^+^, or *Piezo2*^+^ within each subtype. (*F*) Heatmaps showing differential use of wiring regulators *Flrt2* and *Wnt5a* by *Gad2* or *Vglut3* enriched subgroups of serotonin neurons, respectively.

*Gad2*, *Slc17a8* (*Vglut3*), and *Piezo2* are consistently expressed in serotonergic neurons across embryonic and postnatal stages, defining complementary clusters. Specifically, *Gad2* marked DR 1 to 3 and MR3; *Vglut3* marked DR 4 to 5, MR1, MR2, and cDR; and *Piezo2* marked MR4 (Fig. 4*A*). To trace neonatal serotonergic neurons marked by these genes, we used a viral–genetic intersectional strategy. We crossed *Sert-Flp* mice with *Gad2-Cre*, *Slc17a8-Cre*, or *Piezo2-Cre* driver lines, and injected a dual Flp- and Cre-dependent reporter (AAV-Ef1a-Con/Fon-mCherry) into the DR/MR at P0-1 (Fig. 4*B*). We then mapped the whole-brain projections of these subtypes at P28 using D-LMBmapX (15) for axon segmentation and whole-brain registration (*SI Appendix*, Fig. S7*A*_1_ and Dataset S2). Serotonin axons from the neonatal *Gad2*^+^ population predominantly innervated subcortical regions, including the striatum, dorsal thalamus, epithalamus, and most hypothalamic areas. In contrast, neonatal *Vglut3*^+^ serotonergic neurons preferentially targeted cortical regions, consistent with their adult projection pattern (7). Although the *Piezo2*^+^ subtype was relatively sparse, it exhibited dense projections to multiple areas, including the lateral habenula (LHb), the paraventricular thalamic nucleus (PVT), and the olfactory bulbs (Fig. 4*C*). To capture the global organization of these projection patterns, we quantified axon density across all brain regions and clustered them based on their input profiles from the three subtypes. Annotating each region with the *Gad2*^+^:*Vglut3*^+^ density ratio revealed a strong overall segregation of subcortical vs. cortical targets, while substantial local heterogeneity remained within major clusters (Fig. 4*D*_1_ and Dataset S2). Together, these results indicate that the projection pattern is a major organizing axis of serotonergic neuron molecular identity, and that long-range connectivity is associated with early-stage subtype-specific gene expression.

Next, we asked whether the developmental trajectory of local terminal innervation relates to molecularly defined subtypes. Clustering brain regions by serotonin axon innervation dynamics across P0–P14 (Fig. 4*D*_2_ and *SI Appendix*, Fig. S7*A*_2_) revealed three trajectories: i) a P7 surge with continued growth to P14; ii) a P7 peak followed by a drop at P14; and iii) a P7 peak that plateaus through P14. Cortical regions were enriched for (i), whereas subcortical regions more often showed (ii) or (iii) (Fig. 4*D*_2_, *SI Appendix*, Fig. S7*A*_2_, and Datasets S1 and S3). This pattern aligns well with *Vglut3*^+^ vs. *Gad2*^+^ biases, yet each cluster contains a mix of regions and amplitudes, indicating within-cluster diversity. These results indicate a correspondence between molecular subtype identity and local timing of terminal maturation.

To examine when these molecularly defined projection-biased subtypes engage wiring programs, we performed GSEA of GO Biological Process terms related to neuronal development and summarized NES for each subtype per stage (*SI Appendix*, Fig. S8). Consistent with population-level trends and the serotonin innervation wave, axon-guidance, and synapse-assembly gene sets rose across subtypes with a shared P4 to P7 peak (*SI Appendix*, Fig. S8). We then analyzed the top-expressed genes within these categories for each subtype and found that the underlying gene sets were largely distinct (Fig. 4*E*). Three major expression patterns appeared: subtype-specific across time points (*SI Appendix*, Fig. S9*A*), stage-specific across subtypes (*SI Appendix*, Fig. S8*B*), and patterns specific to both subtype and stage (*SI Appendix*, Fig. S9*C*). Thus, while subtypes engage wiring programs on a coordinated timetable, they do so via different genetic modules, supporting region-specific patterns of innervation.

## Discussion

Despite their small numbers, DR and MR serotonin neurons influence mental state in health and disease through widespread, highly divergent axonal projections across the forebrain. However, little is known about how this complex projection architecture is assembled or which genetic programs specify its innervation. In this study, we integrated anatomical and transcriptomic characterization to uncover a coordinated developmental program that establishes the structural and molecular architecture of the forebrain-projecting serotonin system from embryonic to postnatal stages.

Three key features of serotonergic circuit assembly emerged from our analysis. First, the molecular framework underlying mature serotonin neuron subtype identity is already evident by E14.5. This timing is unexpectedly early relative to dopaminergic (26, 27), cortical (28), and habenular (29) lineages, in which embryonic subtype distinctions are detectable but typically consolidate postnatally with adult-marker expression. Our data show that adult subtype-associated molecular features already distinguish serotonergic subpopulations at embryonic stages, with these distinctions becoming more pronounced during postnatal development. This contrasts sharply with dopaminergic neurons (26, 27), in which robust coexpression of adult subtype markers is typically not observed until late postnatal stages. Because most serotonergic axons have not yet reached their forebrain destination by E14.5, the presence of molecularly distinct serotonergic subtypes at this stage, together with their later contribution to complementary projection patterns, suggests that major features of subtype-specific projection architecture are established early through intrinsic developmental programs that operate before extensive target innervation. Although activity-dependent maturation and target-derived signals likely further refine these circuits during postnatal development, our findings support a model in which early serotonergic subtype identity provides an initial framework for subsequent connectivity.

Second, we found that serotonin axon density undergoes a synchronized surge across forebrain targets between P4 and P7, following the peak activation of wiring-related transcriptional programs at P4. The temporal lag between transcriptional activation and anatomical expansion suggests that the molecular machinery required for terminal field assembly is deployed before large-scale growth becomes apparent at the anatomical level. Earlier studies of selected brain regions up to P28 identified two developmental trajectories, both marked by a surge at P7 (14). Yet it has remained unclear whether these patterns generalize across the whole brain or how strongly innervation dynamics differ across targets. Addressing these questions has been difficult due to the lack of tools for cross-age whole-brain registration and for segmenting developing axons. We applied our recently developed deep-learning toolbox for postnatal mouse brains (15) to achieve cross-age whole-brain registration and unbiased axon quantification. Using the same framework, our analyses of tyrosine-hydroxylase–positive axons found no evidence of a comparable brain-wide synchronization (15). Thus, in contrast to catecholaminergic projections, early-postnatal brain-wide innervation synchrony appears specific to serotonergic afferents.

Finally, our analysis revealed markedly heterogeneous regional developmental trajectories of serotonergic terminals, with molecular identity, target region, terminal maturation dynamics, and differentially expressed wiring programs correlated with one another. Consistent with previous analyses of selected brain regions (14, 16), our whole-brain serotonin axon density atlas revealed that by P0, most brain regions have already received serotonin innervation. This suggests that wiring regulators expressed in specific subtypes during embryonic stages likely guide axon pathfinding, whereas those with subtype-specific expression in later stages contribute to regional wiring refinement. We speculate that the highly diverse innervation patterns after P7 across brain regions, including increased, sustained, and decreased density, are jointly modulated by postsynaptic regions. On the other hand, our previous study demonstrated that the DR serotonin neuron subtypes exhibit input–output wiring specificity (6). Because of that, we also speculate the peak of genes related to dendrite development at P7 may contribute to input–output specification together with genes related to synapse assembly. Taken together, our findings support a developmental framework in which serotonergic circuit assembly is organized hierarchically. An early population-wide program establishes a common temporal window for terminal field growth, while subtype-specific molecular programs and target-dependent interactions govern distinct patterns of terminal maturation across projection targets.

A limitation of this study is that cells from multiple animals were pooled before sequencing, preventing formal assessment of sex-specific transcriptional differences at the level of biological replicates. Nevertheless, exploratory analyses based on sex-chromosome marker expression identified only limited sex-associated transcriptional variation beyond expected sex-linked genes (*SI Appendix*, Fig. S10).

The three major ascending neuromodulatory systems—dopamine, norepinephrine, and serotonin—regulate forebrain function primarily through volume transmission. Their axons contain varicosities that serve as neurotransmitter release sites, but synaptic appositions are largely absent (30). Most of what we know about neuron subtype differentiation, subtype-specific wiring, and the molecular mechanisms governing these processes in neuromodulatory systems comes from studies of the midbrain dopamine system (31). In this study, we laid the groundwork for understanding these aspects of the serotonin system, and revealed distinct principles from the dopamine system. Our work also opens avenues for precision intervention in disorders where selective modulation of distinct serotonin circuits may enhance therapeutic outcomes.

## Materials and Methods

### Animals.

For scRNAseq experiments, Sert-Cre mice (MMRRC, Stock #017260-UCD) were crossed with the Ai14 tdTomato Cre reporter mice (JAX Strain 7914). For subtype-specific axon quantification, *Gad2-Cre*, *Vglut3-Cre,* or *Piezo2-Cre* mice were crossed with *Sert-Flp* mice for intersectional labeling by P0-1 injection of a dual Flp- and Cre-dependent reporter (AAV-Ef1a-Con/Fon-mCherry). Both male and female mice on a CD1 and C57BL/6J mixed background were used unless specified. For whole brain quantification of serotonin axon density in P0, P4, and P7 brains, serotonin axons in wild-type mouse brains were visualized by anti-Tph2 staining. For P10 and P14, GFP was stained in Tph2-eGFP mouse brains (5). We initially attempted anti-Tph2 immunostaining across all developmental stages; however, in whole cleared brains older than P10, the anti-Tph2 antibody showed insufficient tissue penetration and incomplete labeling of serotonergic axons. The Tph2-eGFP line was therefore used for P10 and P14 samples to ensure robust whole-brain visualization of serotonergic projections. Both male and female mice were used. Sert bacTRAP mouse (JAX Strain 028620) embryos at embryonic day 14.5 were used for fluorescent in situ hybridization. For details about the mice strains used and housing conditions, see *SI Appendix*, *Materials and Methods*.

### Stereotaxic Surgeries.

Dual Flp- and Cre-dependent reporter pAAV-Ef1a-Con/Fon-mCherry was a gift from Karl Deisseroth (Stanfrod University, CA) and INTRSECT 2.0 Project (Addgene viral prep # 137132-AAV8; http://n2t.net/addgene:137132; RRID: Addgene_137132). AAVs was injected to DR and MR using the lambda as the reference point. We measured coordinates for DR injection at −2.0 AP, 0.0 ML and performed two injections (250 nL) one at −2.50 DV and the other at −2.75 DV. For virus injection in the MR (100 nL), a single injection at −1.8 AP, 0.0 ML, −2.7 DV was performed. For details about the surgical conditions, see *SI Appendix*, *Materials and Methods*.

### Tissue Clearing and Immunostaining.

Mice of various postnatal stages (P0 to P14) were transcardially perfused with PBS followed by perfusion with 4% PFA (paraformaldehyde). Brains then were carefully removed from the skull and postfixed overnight at 4 °C and cleared using a protocol modified from Adipo-clear (32, 33). Samples were imaged on the Miltenyi Biotec UltraMicroscope BlazeTM light sheet microscope (Miltenyi Biotech) in the axial position. Brains with superficial damage or internal cracks were excluded from subsequent analysis. For details about tissue clearing, immunostaining, and imaging settings, see *SI Appendix*, *Materials and Methods*.

### 2D and 3D Whole Brain Registration and Axon Segmentation.

After preprocessing to enhance image quality and normalize images across developmental stages, brains were registered to the developmental atlas using D-LMBmapX (15) and automatically segmented using the approach provided in D-LMBmap (34). Manual annotation of smaller brain regions improved the alignment accuracy in early-stage brains.

After whole-brain axon segmentation and registration to the developmental atlas, axon density was automatically quantified across 12 major brain areas (Allen Brain Atlas Level 5) and 266 brain regions (Allen Brain Atlas Level 8). Fiber tracts, ventricles, raphe nuclei, and regions posterior to the raphe nuclei were excluded from the analysis at each developmental stage. This resulted in a total of 196 qualified regions (Dataset S1). Axon density for each brain region was computed as the total number of axon pixels within the region, divided by the number of voxels in that region, using the D-LMBmapX developmental brain atlas at different stages. Axon density z-scores were calculated for each brain region. Brain regions were clustered using Ward D clustering to generate the heatmap in Fig. 4*D*_2_. For the axon density heatmap in Fig. 4*D*_1_ and Dataset S2, raw axon densities in each brain area were averaged (n = 3 per genotype) and grouped by Ward D clustering. For details about whole brain registration and axon segmentation, see *SI Appendix*, *Materials and Methods*.

### scRNAseq Sample Preparation.

For scRNAseq, brain tissue was collected from transgenic *Sert-Cre;Ai14* mice of mixed sex from developmental days E14.5 - P28. Worthington Papain Dissociation System (Worthington Biochemical Corporation, Cat#:LK003150) was used during tissue preparation according to the manufacturer’s protocol. Single cells sorted based on tdTomato expression and Hoechst staining were processed using 10× Chromium Next GEM Single Cell 3’ HT Reagent Kits v3.1 (Dual Index) (Cat: PN-1000348) to generate single cell/nuclei 3’ gene expression dual index libraries. The samples were then sequenced with the NovaSeq 6000 (Illumina). Detailed protocols are listed in *SI Appendix*, *Materials and Methods*. Sex was subsequently inferred from expression of sex chromosome-linked genes, revealing that most developmental stages contained both male and female cells, whereas the P0 dataset contained only female cells. Because cells from multiple animals were pooled prior to FACS isolation and sequencing, the contribution of individual animals could not be tracked. Therefore, the study was not designed to formally assess sex-dependent transcriptional differences.

### Transcriptomic Data Analysis.

Low quality cells were removed from the dataset. Serotonin neurons were identified based on expression of key serotonergic markers. Each developmental stage dataset integrated to a published Smart-seq2 scRNA^DR&MR^ dataset (7) using Seurat V5 (35), subtype labels were assigned using a k-nearest neighbor approach. Only labels that were recovered in at least 75 of 100 iterations were accepted; all remaining cells were designated as “Unassigned.” Pseudotime assignment, marker gene detection, gene regulatory network prediction and gene set enrichment analysis were performed on the datasets based on these assigned labels. For cross species integration of mouse and human datasets, scAlign (36) was used to optimize the CCA reduction of the combined datasets. For details about scRNAseq and subsequent data processing, see *SI Appendix*, *Materials and Methods*.

### Fluorescent In Situ Hybridization.

Sert bacTRAP mouse line was used as the serotonin neurons are specifically labeled with GFP. Embryos were collected at embryonic day 14.5, fixed in 4% PFA and cryoprotected in 30% sucrose in diethyl pyrocarbonate (DEPC)-treated water. Samples were cryosectioned to 20 mm and stained using multiplexed hybridization chain reaction (HCR) (37) with HCR Gold RNA-FISH kit (Molecular Instruments). Details about probe concentrations and probe-amplifier combinations are listed in *SI Appendix*, *Materials and Methods*.

### Statistical Analysis.

Data are presented as mean ± SD. A *P* value < 0.05 after correction for multiple testing was considered significant unless otherwise stated. For details about statistical tests and corrections for multiple testing used, see *SI Appendix*, *Materials and Methods*.

## Supplementary Material

Appendix 01 (PDF)

Dataset S01 (XLSX)

Dataset S02 (CSV)

Dataset S03 (CSV)

Movie S1. Movie showing a 3D rendering of serotonin neuron axons in select cortical brain regions at developmental stage P4. The selected brain regions and their timestamps are as follows; ACA (0:00), PL (0:21), ORB (0:27), ILA (0:36), AI (0:43).

Movie S2. Movie showing heatmaps of serotonin neuron axon densities across the whole mouse brain at P0, P4, P7 and P14 developmental stages. The heatmaps are depicted from a coronal perspective and the movie proceeds along the anterior-posterior axis.

## Data Availability

Raw sequencing data are available at NCBI’s GEO database (GSE290607) (38). All analysis code used in this study, including subtype label transfer, correlation analyses between subtypes, and scAlign-based dataset integration, is publicly available at https://github.com/kwu-lmb-neurobiology/Serotonin_subtypes (39).
