# Supplementary material for: System-level synchronization and subtype-specific modules govern brain-wide serotonin axon innervation
Source: Proc Natl Acad Sci U S A. 2026 Sep 22;123(39):e2611533123. doi: 10.1073/pnas.2611533123 (PMC13624646; doi:10.1073/pnas.2611533123)
Supplement: Supplementary file 1 [file pnas.2611533123.sapp.pdf]

Supporting Information for
System-level Synchronisation and Subtype-specific Modules Govern Brain wide Serotonin Axon Innervation

Annabel M.J. Adams[a†], Kenneth Lap Kei Wu[a†*], Zhongyu Li[a,b,c], Peiqi Li[c], Hangzhen Pan[c], Zuqi Huang[c], Sara Migliarini[d], Richard Dear[a], Bo Sun[f], Zora Chui-Kuen Chan[a], Jingwen Liang[a], Hongjie Li[f], Petra E. Vértes[e], Massimo Pasqualetti[d], Alina Isakova[g*], Jing Ren[a*].

Correspondence: Jing Ren, Alina Isakova, Kenneth Lap Kei Wu
Email: jren@mrclmb.ac.uk, isakova@stanford.edu, kwu@mrclmb.ac.uk

This PDF file includes:

- Extended Materials and Methods
- Figures S1 to S10
- Tables S1 to S2
- Legends for Movies S1 to S2
- Legends for Datasets S1 to S3
- SI References

Other supporting materials for this manuscript include the following:

- Movies S1 to S2
- Datasets S1 to S3

Materials and Methods

Animals
All experiments performed in the UK involving the use of live animals were performed with respect to the UK Animals (Scientific Procedures) Act 1986. All procedures and experimental designs involving live animals in the UK were approved by the MRC Laboratory of Molecular Biology Animal Welfare and Ethical Review Body (AWERB) under project license PP6471806. Animals were kept in a controlled environment the temperature was kept within a range of 19–23 °C and humidity within a range of 45–65%. Mice were housed in cages in groups of up to five animals. Water and food were provided ad libitum. Lighting was set to a light:dark cycle, with simulated dusk and dawn at 19:00 and 07:00. For scRNAseq experiments, *Sert-Cre* mice (MMRRC, Stock #017260-UCD) were crossed with the *Ai14 tdTomato* Cre reporter mice (JAX Strain 7914). For subtype-specific axon quantification, *Gad2-Cre*, *Vglut3-Cre* or *Piezo2-Cre* mice were crossed with *Sert-Flp* mice for intersectional labelling by P0-1 injection of a dual Flp- and Cre-dependent reporter (AAV-Ef1a-Con/Fon-mCherry). Sert bacTRAP mouse (JAX Strain 028620) embryos at embryonic day 14.5 were used for fluorescent in situ hybridization. Both male and female mice on a CD1 and C57BL/6J mixed background were used unless specified.

Tph2-EGFP knock-in mice were generated in the previous study (1) and housed in the University of Pisa, Italy, at constant temperature (22±1 °C) and maintained on a 12/12 h light/dark cycle, with food and water ad libitum. Experiments involving *Tph2-EGFP* knock-in mice were conducted in accordance with the Ethics Committee of the University of Pisa and approved by the Veterinary Department of the Italian Ministry of Health (Permit number AC179.N.HQV).

Stereotaxic surgeries
Neonatal mice (P0-1) were anesthetised with 5% isoflurane, and mounted on a specially adapted 3D printed mould and gently secured in place (2) with isoflurane maintained at 1 – 1.5 % during surgery. A light source within the transparent mould allows for visualisation of the lambda skull suture through the skin. Using the lambda as the reference point, we measured coordinates for DR injection at -2.0 AP, 0.0 ML and performed two injections (250nl) one at -2.50 DV and the other at -2.75 DV. For virus injection in the MR (100nl), a single injection -1.8 AP, 0.0 ML, -2.7 DV was performed. The time for injections were not to exceed 7 minutes per pup. After all pups which were injected recovered, the litter was returned to the home cage with their mother after no more than one hour had passed.

Viral constructs and Viruses packaging
pAAV-Ef1a-Con/Fon-mCherry was a gift from Karl Deisseroth & INTRSECT 2.0 Project (Addgene viral prep # 137132-AAV8; http://n2t.net/addgene:137132 ; RRID:Addgene_137132).

Tissue clearing and immunostaining
Mice of various postnatal stages (P0-P14) were transcardially perfused with PBS followed by perfusion with 4% PFA (paraformaldehyde). Brains then were carefully removed from the skull and postfixed overnight at 4°C. The following protocol was modified from Adipo-clear (3, 4). Brains were then washed three times in PBS to remove the PFA. Brains are then gradually dehydrated with an increasing methanol (MeOH) gradient: 20%,40%,60%,80%,100%,100% with B1N buffer as the counterpart, with 50-minute incubations per step. For P7 brains and below 1:1 B1N and H20 was used as the MeOH counterpart at 80% step. Brains are then delipidated overnight in 2:1 dichloromethane (DCM): MeOH. The following day, brains are changed to 100% DCM for one hour (two washes) then dehydrated in 100% MeOH for two hours (two washes). Brains are then bleached in 5:1 MeOH and hydrogen peroxide (H_20_2) for four hours. After that, the brains are rehydrated in 60%,40%,20% MeOH/B1N for 50 minutes per step. Samples are then changed into 100% B1N and left overnight. The next day samples are permeabilised in permeabilization buffer for three hours at 37°C (two solution changes). The samples are then changed into PtxwH buffer for three hours at 37°C (two solution changes). For brains of P0, P4 and P7 mice, serotonin projections were labelled by staining with the anti-Tph2 Polyclonal Rabbit IgG antibody (1:1000, Biotechne, Lots: D-2,DIO7289-1,DIO7289-2,DIO7289-3,DIO7289-4 ,Cat: NB100-74555). For brains of P10 and P14 mice, serotonin projections were labelled by the anti-GFP Polyclonal

Chicken Antibody (1:1000, Aves Labs Inc, Lots: GFP917979, GFP3717982, Cat: GFP-1010) staining with the *Tph2-GFP* knock-in mouse line. For Anti-Tph2 staining, the primary antibody is added in PtxwH a fresh 5ml tube and the brain is left to incubate at 37°C with agitation for 7 – 14 days. For Anti-GFP staining, the primary antibody is added in PtxwH and left to incubate at 4°C with agitation for 2 months. After primary staining, the brains are transferred to a fresh 5ml tube with PtxwH and washed five times over the first day at 37°C with agitation. After this, brains are washed for a further two days with two solution changes at 37°C with agitation. For the final wash step, samples are transferred into a new tube of PtxwH. For secondary antibody staining, the brains are incubated in Alexa Fluor® 647 AffiniPure™ Donkey Anti-Chicken IgY (1:1000, Jackson Immuno- Research, Lots:171662,155990,161533, Cat: 711-605-152 or Alexa Fluor® 647 AffiniPure™ Donkey Anti-Rabbit IgG (1:1000, Jackson Immuno- Research, Lots: 171662,155990,161533, Cat: 711-605-152) secondary antibody and PtxwH at 37°C with agitation for four-seven days. To wash of the secondary antibody, the brains are transferred to a fresh 5ml tube with PtxwH and washed five times over the first day at 37°C with agitation. The brains are then washed for a further two days with two solution changes at 37°C with agitation. Brains are again dehydrated with an increasing methanol (MeOH) gradient: 20%,40%,60%,80%,100%,100% with H20 as the counterpart, with 50-minute incubations per step. For brain samples P7 and below, we used B1N/H20 as the counterpart for all steps. The samples are dilapidated overnight in 2:1 DCM:MeOH. The samples are changed to 100% DCM for two hours (two washes) and then into 100% ethyl cinnamate (ECI) for four hours. After four hours and the tissue has become fully transparent, the brains are switched to a fresh tube with 100% ECI.

Light-sheet imaging and 3D whole-brain analysis
Samples were imaged on the Miltenyi Biotec UltraMicroscope BlazeTM light sheet microscope with ImspectorPro 737 Imaging software (Miltenyi Biotech), in the axial position in the 647 channel with a 4x lens, 1x zoom and 3 μm z-step size. Antibody fluorescence was collected from a 640 nm laser and autofluorescence captured from 488 nm illumination. Resulting images were stitched together using TeraStitcher v1.10.12 (5) software. Brains with superficial damage or internal cracks were excluded from subsequent analysis.

3D whole brain registration
Each whole-brain 3D volume in the autofluorescence channel was first pre-processed using a series of image processing operations, including orthogonal rotation, cropping, mirroring, contrast enhancement, N4 bias correction, intensity rescaling, and 3D contrast-limited adaptive histogram equalization. These steps enhanced the original image quality and normalized 3D brain images across developmental stages.

Next, each brain was registered to its corresponding developmental atlas using the 2.5D registration method described in D-LMBmapX (6). Each brain sample was downsampled to a fixed resolution (320*456*528). Given that brain tissue in early developmental stages (P0 to P7) is highly susceptible to deformation, we incorporated brain region constraints into the 2.5D registration process to ensure consistent alignment between experimental brains and the developmental atlas. Specifically, six major brain regions—CB, BS, CP, HPF, CTX, and OLF—were automatically segmented using the approach provided in D-LMBmap (7).

Additionally, to improve alignment accuracy in early-stage brains, we manually annotated ten smaller brain regions within the brainstem, including the Red Nucleus (RN), Dorsal part of the Lateral Geniculate Complex (LGd), Medial Habenula (MH), Lateral Habenula (LH), Fasciculus Retroflexus (fr), Anterior Commissure Olfactory Limb (aco), Anterior Commissure Temporal Limb (act), Basolateral Amygdalar Nucleus (BLA), Ventral part of the Lateral Geniculate Complex (LGv), and Anterior Pretectal Nucleus (APN).

3D axon segmentation for whole brain serotonin projection profiling
Each stage includes at least three intact half brains for statistical analysis. Image volumes were processed and analysed with D-LMBmap (7) and D-LMBmapX (6). To train the model, we first selected 150 'artefact' and 209 'axon' cubes from E14.5 to P7 and automatically annotated them following the pipeline in D-LMBmap. These annotated 3D cubes were then used to fine-tune the

neuron foundation model provided by D-LMBmapX. The neuron foundation model in D-LMBmapX demonstrated strong generalization capabilities through self-supervised pre-training. We further refined the model using the automatically annotated cubes. Finally, whole-brain axons from E14.5 to P14 were automatically segmented.

After whole-brain 3D axon segmentation and registration to the developmental atlas, axon density was automatically quantified across 12 major brain areas (Allen Brain Atlas Level 5) and 276 brain regions (Allen Brain Atlas Level 8). Fiber tracts, ventricles, raphe nuclei, and regions posterior to the Raphe nuclei were excluded from the analysis at each developmental stage. This resulted in a total of 196 qualified regions (SI Appendix, Table S1). Axon density for each brain region was computed as the total number of axon pixels within the region, divided by the number of voxels in that region, using the D-LMBmapX developmental brain atlas at different stages. Axon density z-scores were calculated for each brain region. Brain regions were clustered using Ward D clustering to generate the heatmap in Fig $4D_2$.

scRNAseq sample preparation
For scRNAseq, brain tissue was collected from transgenic *Sert-Cre;Ai14* mice of mixed sex from developmental days E14.5 - P28. A minimum of five brains and a maximum of eight brains were used per preparation batch. Unless otherwise stated, all solutions were kept on ice and with O2/CO2 bubbling throughout. Worthington Papain Dissociation System (Worthington Biochemical Corporation, Cat#:LK003150) was used during tissue preparation. For the first stages of sample preparation S buffer was used to incubate and slice the brain tissue. The S buffer was first made up to a 10X stock; (in g/500ml: Choline Chloride 76.79, Potassium Chloride 0.93, Calcium Chloride 0.38, Magnesium Chloride Hexahydrate 7.12 , Monosodium Phosphate 0.77, Sodium Ascorbate 1.29, Sodium Pyruvate 0.33, made up to 500ml with Milli-Q® Ultrapure Water) then the 1X working solution was made up with the following additions; (in g/500ml: Sodium Chloride 36.52, Potassium Chloride 0.93, Calcium Chloride 1.47, Magnesium Chloride Hexahydrate 1.32, Monosodium Phosphate 0.77,Sodium Ascorbate 1.29, Sodium Pyruvate 0.33, 50ml S buffer 10X stock and 450ml with Milli-Q® Ultrapure Water). The tissue was sectioned on a vibratome in S buffer solution with added Kynurenic acid (KA) 0.5m/l, Actinomycin D (Act D) (Adooq Biosciences, Cat:A13239) 0.05M/L and Tetrodotoxin (TTX) 1mM. The midbrain tissue containing DR and MR was sectioned into 250μm slices which were left to incubate for one hour at 34°C in 5ml oxygenated R solution/papain/Deoxyribonuclease 1. After incubation, the DR and MR brain slices were transferred back on ice and micro dissected based on anatomical markers and the fluorescence signal from the TdTomato expression. For the subsequent steps in tissues preparation, R Solution was used. R Solution was first made up to a 10X stock; (in g/500ml: Sodium Chloride 36.52, Potassium Chloride 0.93, Calcium Chloride 1.47, Magnesium Chloride Hexahydrate 1.32, Monosodium Phosphate 0.77, Sodium Ascorbate 1.29, Sodium Pyruvate 0.33 and made up to 500ml with Milli-Q® Ultrapure Water) then the 1X working solution was made up with the following additions; (in g/500ml: Sodium Bicarbonate 1.05,Glucose 2.25, 50ml R buffer 10X stock and 450ml Milli-Q® Ultrapure Water). The sections were then removed from bubbling O2/CO2 and transferred to 500μl oxygenated R solution. The sections where then mechanically dissociated for 20 minutes using gentle repeated pipetting with glass pipettes of an increasingly narrow diameter. The supernatant was removed, and the cells were resuspended in 300μl LI buffer (made up by adding, 300μl of HI buffer, 150μl of Dnase (Worthington Dissociation Kit vial 2) to 2.7ml of Oxygenated R solution) and then transferred to a new tube with 500μl HI buffer (made up by adding 32ml Earle's balanced salt solution to one vial of ovomucoid inhibitor (Worthington Dissociation Kit)). The solution was then spun again at 350 x g, 4°C for 10 minutes. The supernatant was discarded, and the pellet resuspended in 2 ml oxygenated R solution containing 5ug/ml Hoechst stain. Before flow cytometry, the suspended tissue was passed through falcon 5ml 35μm cell strainer tubes. The cells were sorted using a chilled SONY Synergy cell sorter, with the collection gate based on Hoechst detection with the 375nm laser and TdTomato with the 561nm laser. The cells were sorted into 35 μl of oxygenated R solution and kept on ice until library preparation.

After flow cytometry, samples were prepared for gel bead-in-emulsion (GEM) partition and cDNA library construction through the Chromium Single Cell Gene Expression High Throughput (HT) workflow to the specifications outlined by 10X genomics. The 10X Chromium Next GEM Single Cell

3' HT Reagent Kits v3.1 (Dual Index) (Cat: PN-1000348) and Chromium controller was used to generate single cell/nuclei 3’ gene expression dual index libraries from our samples. For the scRNAseq samples a PCR amplification of 13 cycles for cDNA libraries were used, for snRNAseq samples, 19 cycles were used. The samples were then sequenced with the NovaSeq 6000 (Illumina).

Transcriptomic data processing and analysis

For developmental scRNAseq samples from E14.5 to P28, the following quality control measure were performed. Prior to doublet removal, basic quality control was performed, removing cells with fewer than 300 genes detected and genes detected in fewer than three cells were also removed. Individual sequencing batches were run through DoubletFinder doublet detection package (8), with a doublet formation rate assumed to be 0.8%. Cells identified as doublets were then removed. The data was then filtered based on the expression of key serotonergic markers. To be included, cells must express at least one count of *Tph2* and greater than one count of *Sert*. Furthermore, cells which found to have a percentage expression of mitochondrial genes above 15% were excluded. Finally, low complexity cells with fewer than 10,000 counts detected were removed.

Transcriptomic dataset Integration and label transfer

Integration was performed using Seurat V5 (9). Transcriptomes were normalized by log normalization and integrated with the SmartSeq scRNA$^{DR\&MR}$ dataset using CCA integration. A k-nearest neighbour approach was used to assign labels to the query data based on proximity of query data to reference cell types on the tSNE plot from the first 30 PCA components. Briefly, either the reference or query data was randomly down sampled to the same number of cells to reduce bias from over-representation. Query cells were assigned as the most common label from reference cells within the 30 nearest neighbours, except for query cells that had < 3 reference cells among their 30 nearest neighbours in which case a label would not be assigned. This process was repeated until all query cells were evaluated for 100 - 110 cycles. Labels that were stable for > 75% of cycles were adopted as the final identity of the query cell, otherwise no label was assigned.

To assess if the integration results depended on the strategy used, developmental stage scRNA$^{DR\&MR}$ datasets were integrated with the SmartSeq scRNA$^{DR\&MR}$ dataset using linear latent space methods RPCA, JPCA, and Harmony (10), neighbourhood alignment methods fastMNN (11), Scanorama (12), BBKNN (13) and generative modelling method scVI (14). RPCA, JPCA, fastMNN and Harmony were implemented through the Seurat IntegrateLayers() function, Scanorama, BBKNN and scVI were downloaded from the GitHub link provided in the respective publications and run in Python using raw counts matrices exported from Seurat and re-built as Anndata objects in Scanpy. All integration methods were performed using default parameters. UMAP coordinates were calculated for each integrated object. Subtype labels were assigned to developmental stage cells in the integrated object based on the knn-based method described above. The percentage of cells that could be assigned to a cluster at each stage along with the percentage of assigned cells with the same label as that assigned by Seurat CCA integration were calculated.

Cells in developmental stage scRNA$^{DR\&MR}$ datasets were clustered in an unsupervised manner with Seurat v5 FindClusters() function. A contour encapsulating 80% of cells of each unsupervised cluster was generated with a two-dimensional kernel density estimation function.
To compare subtype identitites propagated from the adult stage to natural clusters, Jaccard index was calculated for each pair of subtype identities at each developmental stage. Additionally, Spearman correlation was calculated for each pair of subtypes identities using the union of the top 50 marker genes for each subtype from unsupervised clustering and transferred subtype labels. Correlation was not calculated for clusters with less than 3 cells. FDR was estimated by calculating the correlation across 1000 sets of randomly permutated cell cluster labels.

For cross species integration of mouse and human datasets, scAlign (15) was used to optimize the CCA reduction of the combined datasets. A k nearest neighbour approach (described above) was used to transfer projection-defined subtype labels from mouse E14.5 scRNA$^{DR\&MR}$ dataset to *Sert*$^{+}$/*Tph2*$^{+}$ neurons in a human embryonic whole brain transcriptomic dataset (16).

Pseudotime trajectory construction

To obtain a trajectory, single cell RNA seq datasets from developmental stages E14.5 to P28 were further filtered for percentage mitochondrial gene expression <2%, cells whose DR-MR identity or subcluster identity according to Ren, 2019 could not be confidently inferred by KNN, as well as cells in cDR and MR-4 which were transcriptomically highly distinctive, were excluded from trajectory analysis. Data was normalized using NormalizeData function and variations in percentage mitochondrial gene expression and the number of RNA molecules detected per cell (nCount_RNA) were regressed out using ScaleData function in Seurat V5. Cells were plotted in a UMAP computed from the first 30 principal components (PCs). Pseudotime was assigned to each cell based on a principal curve fitted through the UMAP using Slingshot (17). Average pseudotime values of each projection-defined serotonin neuron subtype across the eight developmental stages investigated in this study are summarized in Table S2.

Bootstrapping for marker gene finding

Markers for cells identities within each developmental stage were found using the FindAllMarkers function from Seurat v5 adapted for Bootstrapping. A subset of 70% of cells were sampled from the data and significant markers (adjusted p value = < 0.05) were identified by FindAllMarkers within the subset. The data was resampled with replacement and markers were found across 100 iterations. The markers were then filtered so that only genes found to be significant in 90% of FindAllMarkers iterations were retained. Further, the genes were subsequently filtered so that they must be expressed in at least 50% of the cell population of which they are found to be a marker and so that their average $\log_2$ fold change must be ≥ 1. The genes were then ranked by their average $\log_2$ fold change, where genes from the top ranked markers were selected for further analysis.

Euclidean distance measurement between marker expression profiles

A list of top 10 markers for each subtype of interest as found by Bootstrapping were first filtered for genes that were detected in all developmental stages. For subtypes that had fewer than 10 markers identified by Bootstrapping all the qualified markers were used. Gene expression dotplots were then generated for all developmental stages based on this list using Seurat V5. Data on average expression and percentage of cells expressing each gene was extracted from the dot plots and then vectorized for calculation of Euclidean distances. The total distance for each pair of subtypes was defined as sum of the Euclidean distance for average expression and that for percentage cells expressing the gene. This was plotted as a colour coded matrix.

Analysis of regulons using SCENIC

The python implementation of SCENIC (pySCENIC) (18) was used to detect the gene regulatory networks within serotonin neurons across the 8 developmental stages. Decontx processed and doublet removed counts were used as the input. SCENIC was run with default parameters using the mm9 reference for transcription factor binding sites (https://resources.aertslab.org/cistarget/databases/mus_musculus/mm9/) and cisTarget motifs (https://resources.aertslab.org/cistarget/motif2tf/). Regulon area under the curve (AUC) scores were transformed into z-scores and plotted.

Gene set enrichment analysis

To discover when different subclusters engaged wiring programmes, average log2 fold change of genes was calculated for each subtype and developmental stage combination. gseGO() function from R package clusterProfiler (19) was used to calculate the normalized enrichment score (NES) and false discovery rate (FDR) for processes in GO-Biological Processes (GO-BP). NES and FDR of gene sets relevant to neuronal function including axon guidance (GO:0007411), synapse assembly (GO:0007416), dendrite development (GO:0016358), neurotransmitter transport (GO:0006836), G protein-coupled receptor signaling pathway (GO:0007186), cell-cell adhesion via plasma-membrane adhesion molecules (GO:0098742, abbreviated as “cell-cell adhesion (membrane molecules)”), synaptic transmission glutamatergic (GO:0035249), and neuron migration (GO:0001764) are presented.

For NES plots of individual clusters in SI Appendix, Fig S4, Subclusters that did not contain more than 30 cells in any developmental stage were excluded from GSEA, this included DR-1, DR-4 and cDR clusters. Missing data points indicate that the subcluster did not satisfy the minimum geneset size requirement (10 genes) for gseGO for that GO term. Filled circles indicate FDR < 0.001.

Male and female cell assignment

Cells in $scRNA^{DR\&MR}$ datasets (stages E14.5 – P10) and $snRNA^{DR\&MR}$ datasets (P14 and P28) were labelled as male and female origin based on expression of the y-linked gene Eif2s3y. Differential gene expression was determined using Seurat v5 FindMarkers() function using a Wilcoxon Rank Sum test. Genes were considered significant if the adjusted p-value was less than 0.05 and the absolute value of average log2 fold change was greater than 1.

Fluorescent in situ hybridization

Sert bacTRAP mouse line was used as the serotonin neurons are specifically labelled with GFP. 40-probe probe sets were generated for HCR (20) (Molecular Instruments). Heads were collected from embryonic day 14.5 Sert bacTRAP mice, washed twice in ice cold phosphate buffered saline (PBS) and fixed in 4% paraformaldehyde (PFA) for 12–24 hr in 4°C, cryoprotected in 30% sucrose in diethylpyrocarbonate (DEPC)-treated water for 24–48 hr. Head were then embedded in Optimum Cutting Temperature medium (OCT, Tissue Tek) and stored at –80°C until sectioning.

20 μm sagittal sections spaced every 100 μm were obtained for HCR-FISH using HCR HiFi RNA-FISH kit (Molecular Instruments). Sections were re-fixed in 4% PFA, permeabilized in a 50%, 70% and 100% ethanol series. Hybridization was carried out overnight at 37°C with a probe concentration of 0.2 μl probes per 100 μl hybridization buffer. Amplification was carried out as recommended by Molecular Technologies. Imaging was performed with a Leica Stellaris 5 confocal microscope at 20X. Tissue sections were aligned based on morphology. Position of double labelled cells were manually recorded.

Histology and confocal imaging

Animals were perfused transcardially with PBS followed by 4% PFA. Brains were dissected, post-fixed in 4% PFA for 12–24 hr in 4°C, then cryoprotected by placing in 30% sucrose for 24–48 hr. Brains were then embedded in OCT and stored at –80°C until sectioning.

For data generated in Figure 4 to examine serotonin axonal projections, we performed anti-RFP staining on 40 μm sections, spaced every 200 μm. Brain slices were incubated with primary antibodies rabbit anti-RFP (1:1000, Rockland Lots:48710, Cat: 600-401-379) for 24 hr at 4°C and secondary antibodies for 4 hrs in room temperature. Sections were mounted and coversliped with Fluorogel (Electron Microscopy Sciences). After that, one hemisphere of each brain slice was then imaged using a Leica Stellaris 5 confocal microscope at 20X.

2D axon segmentation and brain region registration

For histology and confocal imaging of 2D brain slices, axons were automatically segmented in the RFP channel. To obtain axon segmentation results, we directly binarized the signals in RFP channel, binarization thresholds were set ranging from 30 to 50 in grayscale. All binarized axons were then skeletonized using the skeletonization step in D-LMBmap. For 2D brain slices in the DAPI channel, pre-processing steps—including orthogonal rotation, cropping, mirroring, and contrast enhancement—were applied to generate intact 2D coronal-view slices. Each 2D slice was then matched to the most similar section in the corresponding 3D brain atlas using the 2D-to-3D localization pipeline in D-LMBmapX. Subsequently, each 2D slice was registered to the corresponding section in the 3D atlas using the ANTs method. Finally, similar to 3D whole-brain profiling, serotonin axon density was automatically quantified across all brain regions captured in the 2D slices.

Axon densities from corresponding sections of 3 mice per genotype were averaged to generate representative spatial maps of axon density in Figure 4C. For the axon density heatmap in Figure $4D_1$ and Dataset S2, raw axon densities in each brain area were averaged (n = 3 per genotype). Regions were grouped by Ward D clustering.

Quantification and statistical analysis

The quality of integration and subtype label transfer by scAlign was evaluated by computing the Spearman correlation between the subtype specific expression pattern of the common top 1000 highly variable genes of the two datasets. Statistical significance for each pair of subtypes between the two species was assessed by comparison to Spearman correlation coefficient from 1000 rounds of shuffling of the gene names of the common genes. A p value < 0.05 after correction for multiple testing using Bonferroni's method was considered significant.

Axon densities were compared across all stages using a one-way ANOVA, followed by Dunnett's post hoc test for comparisons against P0 ($n = 3$ per group). $p < 0.05$ was considered significant.

Enrichment FDR from gseGO() was corrected for multiple testing using Benjamini-Hochberg correction.

Figures

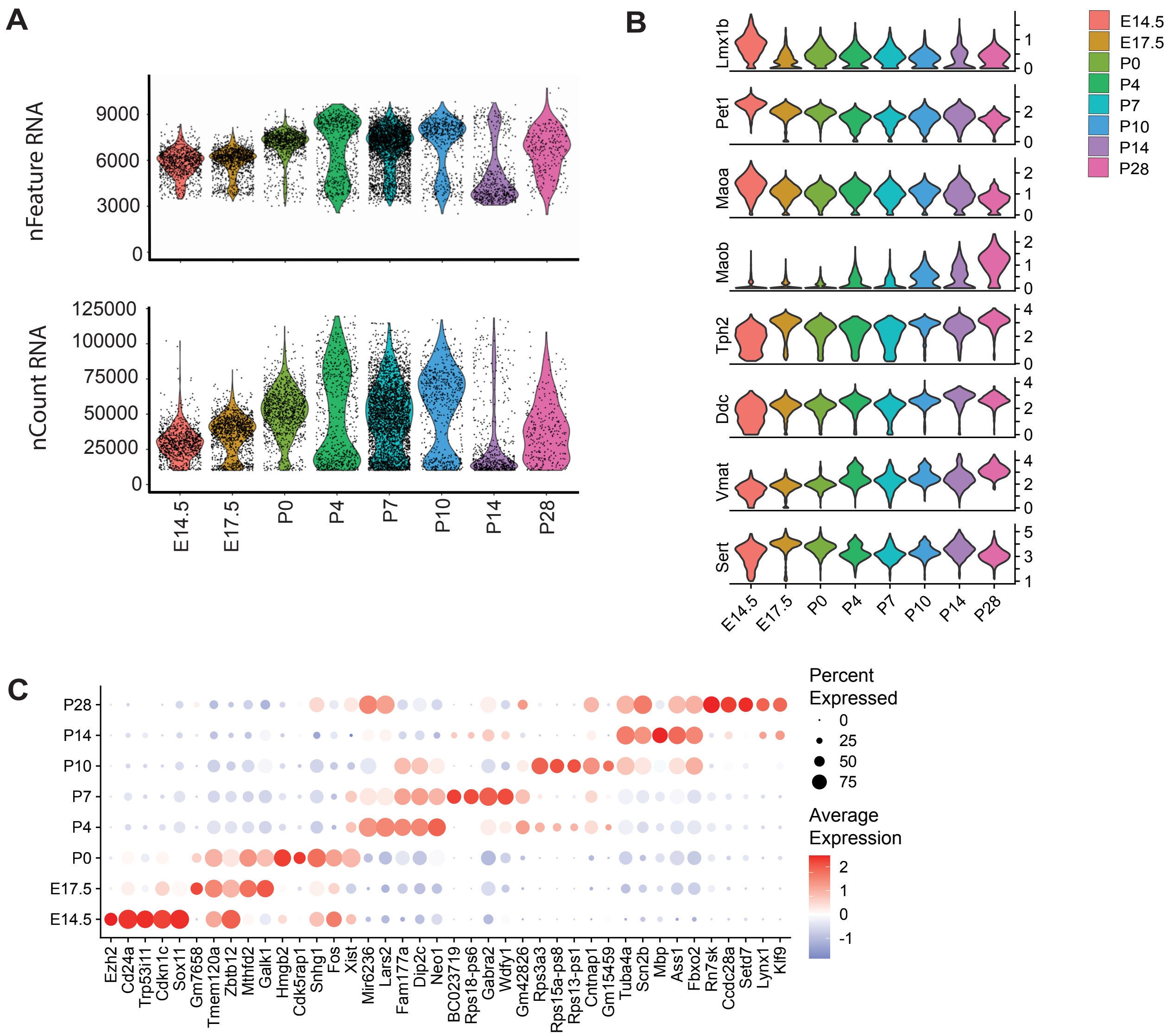

Fig. S1. Technical characteristics of scRNA-seq experiments, related to Figure 2.

A) Violin plots showing the number of genes detedted per cell (nFeature_RNA) and number of reads per cell mapping to exons (nCount_RNA) in qualified serotonin neurons across eight developmental scRNA-seq datasets.

B) Violin plots showing the expression profiles of key genes in developmental scRNA$^{DR\&MR}$ datasets.

C) Dotplot showing top 5 marker genes for each developmental stage in developmental scRNA$^{DR\&MR}$ datasets.

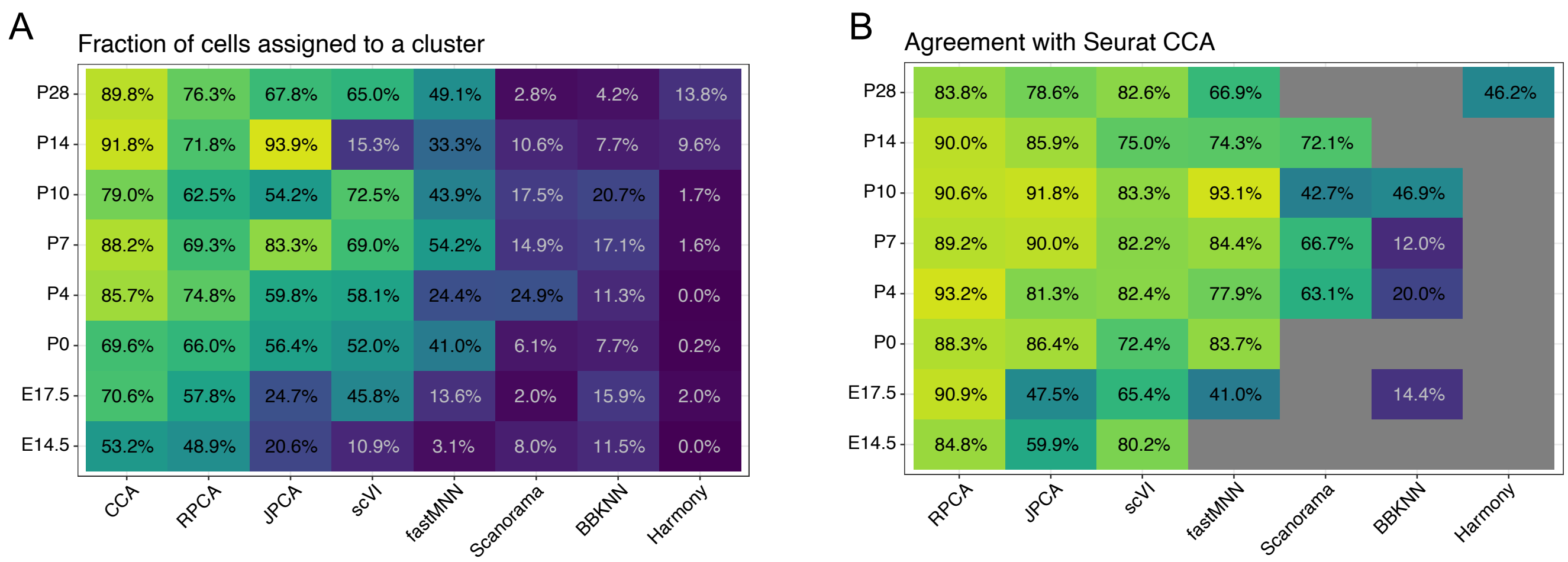

Fig. S2. Comparison between integration methods, related to Figure 3.

A) Heatmap showing the percentage of cells within developmental stage scRNA$^{DR\&MR}$ datasets assigned with a subtype label after integration with the Smart-seq adult$^{DR\&MR}$ dataset using different integration methods. The percentage of cell assignment by Seurat CCA integration is shown in the leftmost column.

B) The percentage of cells assigned to the same subtype as Seurat CCA integration in various developmental stage and integration method combinations. Subtype overlap was not evaluated in cases where cell assignment was less than 10% (shown in grey).

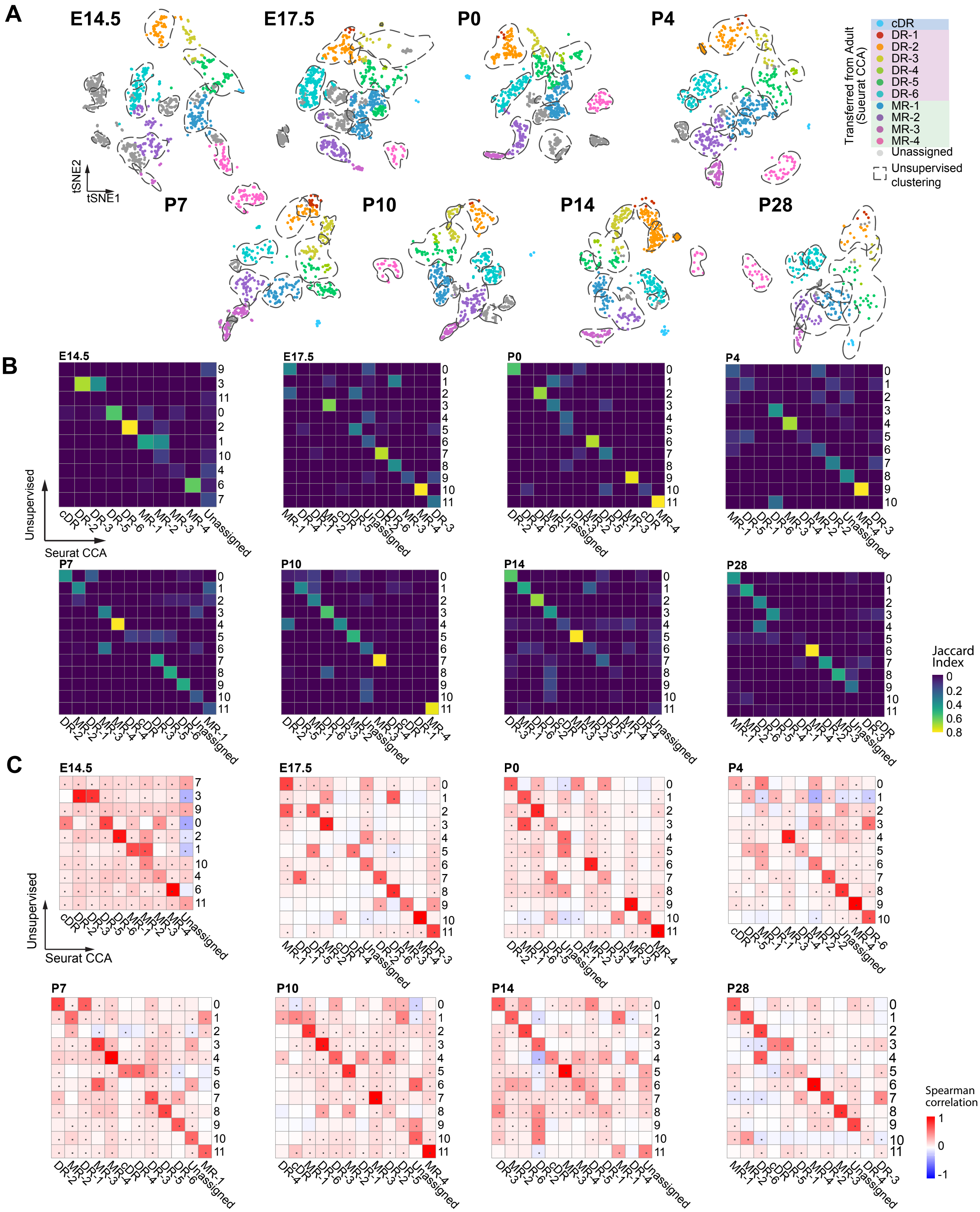
A
E14.5
E17.5
P0
P4
P7
P10
P14
P28
tSNE2
tSNE1
Transferred from Adult (Sueurat CCA)
cDR
DR-1
DR-2
DR-3
DR-4
DR-5
DR-6
MR-1
MR-2
MR-3
MR-4
Unassigned
Unsupervised clustering
B
Unsupervised
Seurat CCA
Jaccard Index
0
0.2
0.4
0.6
0.8
C
Spearman correlation
1
0
-1

Fig. S3. Comparison between transferred subgroup labels and innate transcriptional structures in developmental data, related to Figure 3.

A) tSNE plot of single-cell transcriptomic profiles of the serotonin neuron subtypes in scRNA$^{DR\&MR}$ datasets showing overlap between unsupervised clusters and subtype labels transferred from Smart-seq2 adult$^{DR\&MR}$ dataset after integration. Cells are coloured according to subtype labels transferred from Smart-seq2 adult$^{DR\&MR}$ dataset after Seurat CCA integration. Contours encapsulate 80% of cells of each unsupervised cluster.

B) Jaccard index shows similarity between cluster labels transferred from Smart-seq2 adult$^{DR\&MR}$ dataset and unsupervised clusters in developmental stage scRNA$^{DR\&MR}$ datasets.

C) Spearman correlation between unsupervised and transferred clusters calculated from top 50 marker genes of each cluster in both methods. Dots indicate FDR < 0.05.

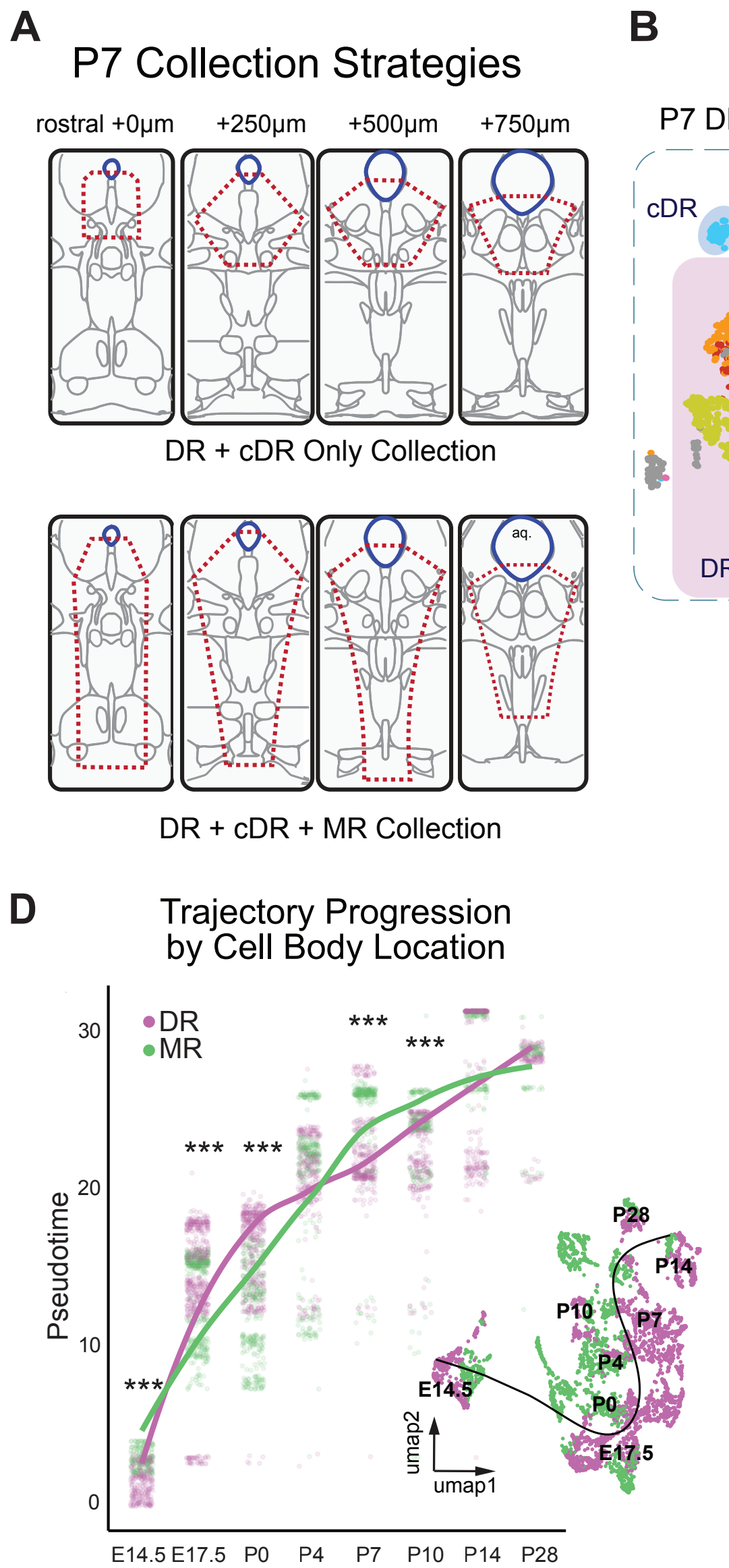
A
P7 Collection Strategies
rostral +0µm
+250µm
+500µm
+750µm
DR + cDR Only Collection
aq.
DR + cDR + MR Collection
B
P7 DR + cDR Only
cDR
DR
MR
cDR
DR-1
DR-2
DR-3
DR-4
DR-5
DR-6
MR-1
MR-2
MR-3
MR-4
Unassigned
C
DR
MR
MR-1
cDR
Unassigned
100
80
60
40
20
0
Combined
DR + cDR Only
DR + cDR + MR
D
Trajectory Progression
by Cell Body Location
DR
MR
Pseudotime
30
20
10
0
***
E14.5
E17.5
P0
P4
P7
P10
P14
P28
umap2
umap1

Fig. S3. Technical characteristics of P7 DR only dataset and pseudotime profile of DR and MR development, related to Figure 3.

A) Schematic showing strategy for collecting “DR + cDR only” versus “DR + cDR + MR” tissue from P7 mouse brain sections.

B) tSNE plots of single-cell transcriptomic profiles of the serotonin neuron subtypes in P7 DR + cDR only scRNA$^{DR\&MR}$ dataset. Cells are coloured according to subtype labels transferred from Smart-seq2 adult$^{DR\&MR}$ dataset after integration.

C) Bar graph showing relative proportion of cells mapped to DR, cDR, MR and MR-1 clusters in P7 “DR + cDR only” and “DR + cDR + MR” datasets, as well as the percentage in the combined dataset.

D) Cell body location (DR and MR) of single cells was inferred based on their clusters. Analysis of pseudotime difference between DR and MR cells revealed rapid maturation of DR serotonin neurons at embryonic stages that was superseded by MR serotonin neurons in the early postnatal stage. *** indicate $p < 0.001$ between pseudotime of DR and MR cells.

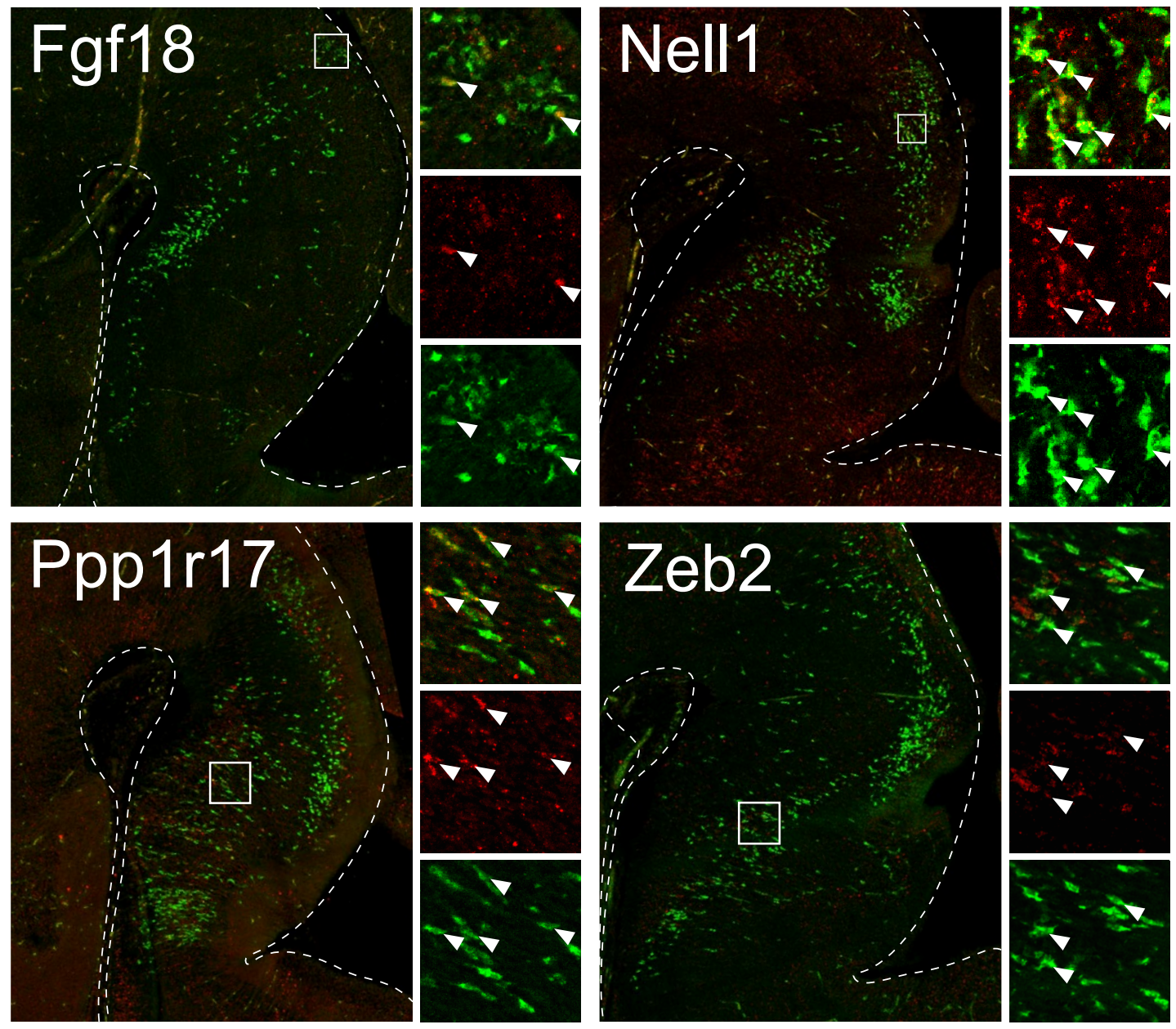

Fig. S4. Spatially segregated expression of subtype specific markers in DR and MR, related to Figure 3.

Spatial expression patterns of subtype markers, Fgf18, Nell1, Ppp1r17 and Zeb2. Sert positive cells are in green with mRNA of the indicated marker gene in red. Inset panels show magnified views of boxed area. White arrows point Sert positive cells expressing the marker gene.

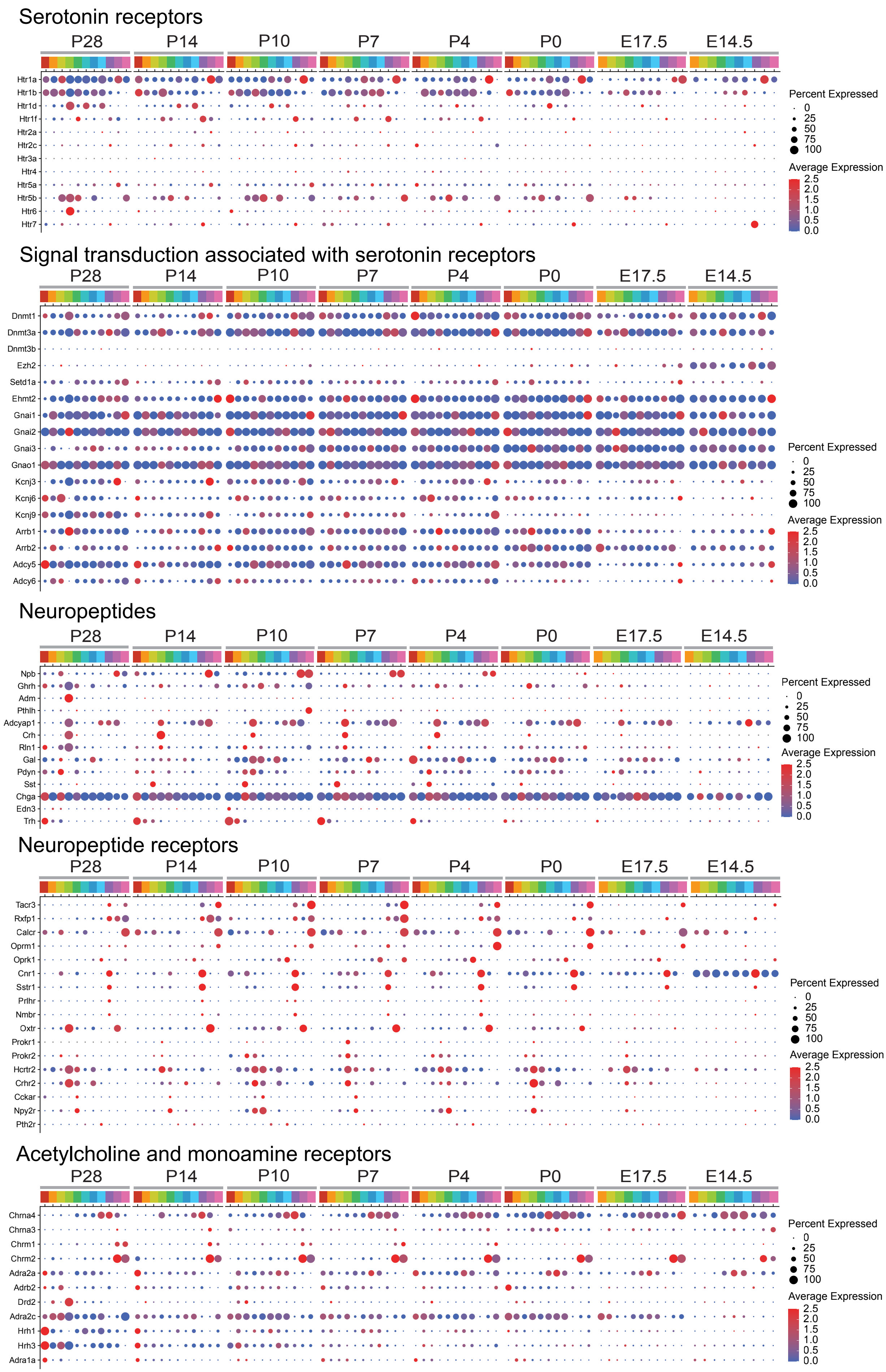
Serotonin receptors
P28
P14
P10
P7
P4
P0
E17.5
E14.5
Htr1a
Htr1b
Htr1d
Htr1f
Htr2a
Htr2c
Htr3a
Htr4
Htr5a
Htr5b
Htr6
Htr7
Percent Expressed
0
25
50
75
100
Average Expression
2.5
2.0
1.5
1.0
0.5
0.0
Signal transduction associated with serotonin receptors
Dnmt1
Dnmt3a
Dnmt3b
Ezh2
Setd1a
Ehmt2
Gnai1
Gnai2
Gnai3
Gnao1
Kcnj3
Kcnj6
Kcnj9
Arrb1
Arrb2
Adcy5
Adcy6
Neuropeptides
Npb
Ghrh
Adm
Pthlh
Adcyap1
Crh
Rln1
Gal
Pdyn
Sst
Chga
Edn3
Trh
Neuropeptide receptors
Tacr3
Rxfp1
Calcr
Oprm1
Oprk1
Cnr1
Sstr1
Prlhr
Nmbr
Oxtr
Prokr1
Prokr2
Hcrtr2
Crhr2
Cckar
Npy2r
Pth2r
Acetylcholine and monoamine receptors
Chrna4
Chrna3
Chrm1
Chrm2
Adra2a
Adrb2
Drd2
Adra2c
Hrh1
Hrh3
Adra1a

Fig. S5. Expression of genes related to serotonergic and neuropeptidergic transmission across development, related to Figure 3.

Dot plots showing expression of serotonin receptors, serotonin receptor-associated signal transduction proteins, neuropeptides, neuropeptide receptors, acetylcholine and monoamine receptors in each serotonin neuron subtype across developmental stages.

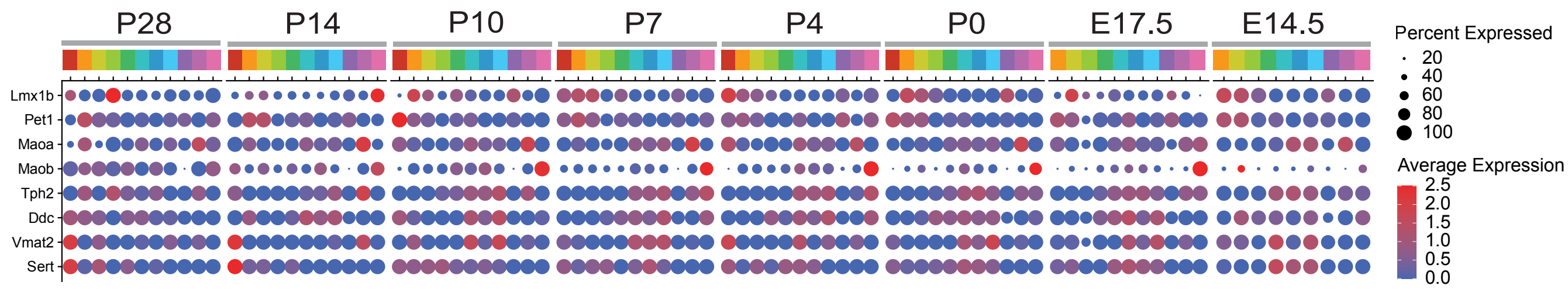

Fig. S6. Expression of key genes related serotonergic system, related to Figure 3.

Dot plot showing subtype dependent expression of key genes related to serotonergic system. See Fig S1B for their stage-dependent expression profile.

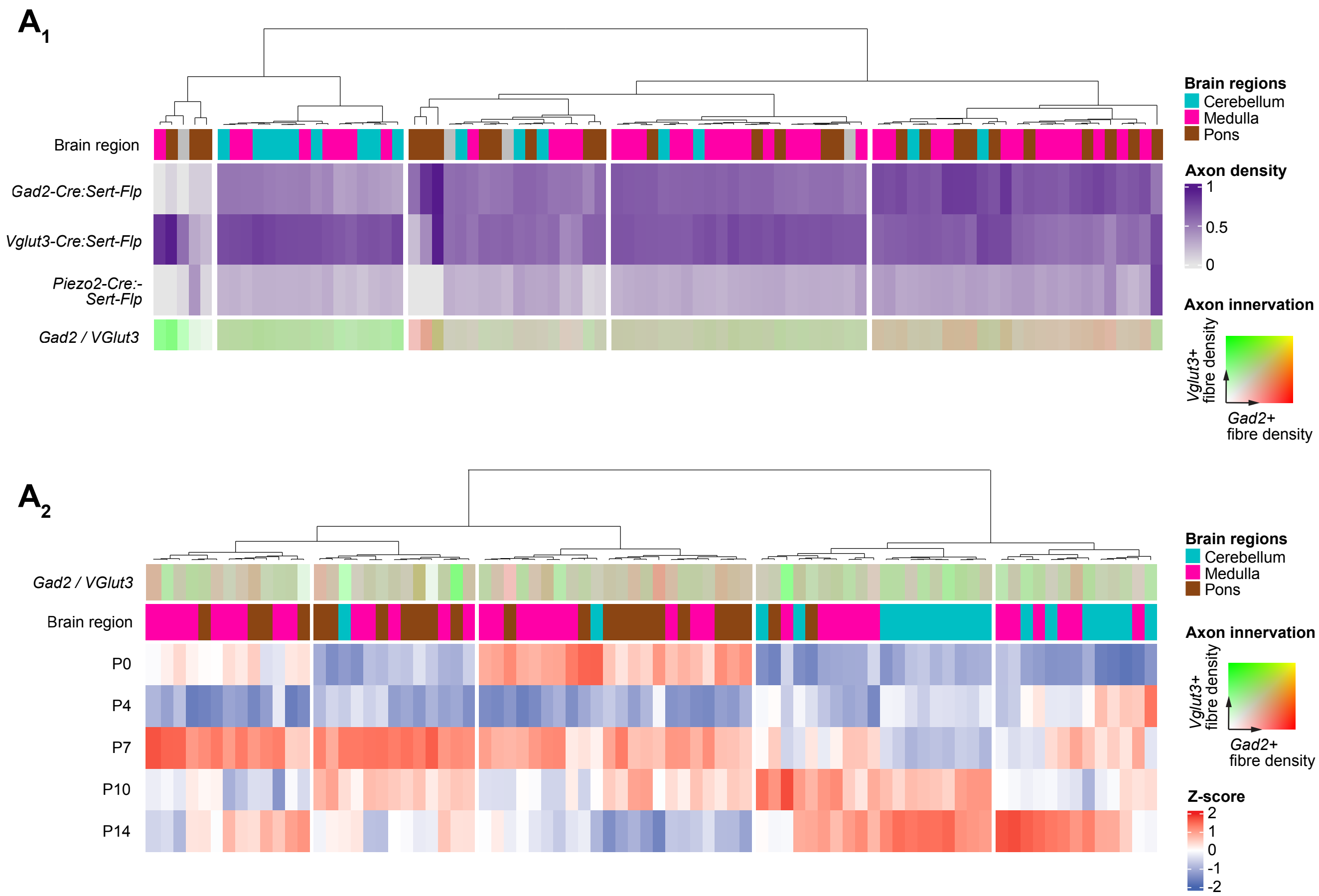

Fig. S7. Serotonin axon densities in hindbrain and cerebellum, related to Figure 4.

A_1) Heatmap of density of *Vglut3*⁺, *Gad2*⁺ and *Piezo2*⁺ serotonin neurons in hindbrain and cerebellar regions at P28. Axon innervation labels *Gad2 / Vglut3* shows the relative density of *Vglut3*⁺ and *Gad2*⁺ axons in each brain region as a gradient. Regions with *Piezo2*⁺ serotonin neuron density z-score > 1 are labelled as *Piezo2* high.

A_2) Heatmap of the developmental profile for serotonin neuron innervation. Axon densities are normalized per brain region. Axon innervation labels for each brain region are inherited from A_1.

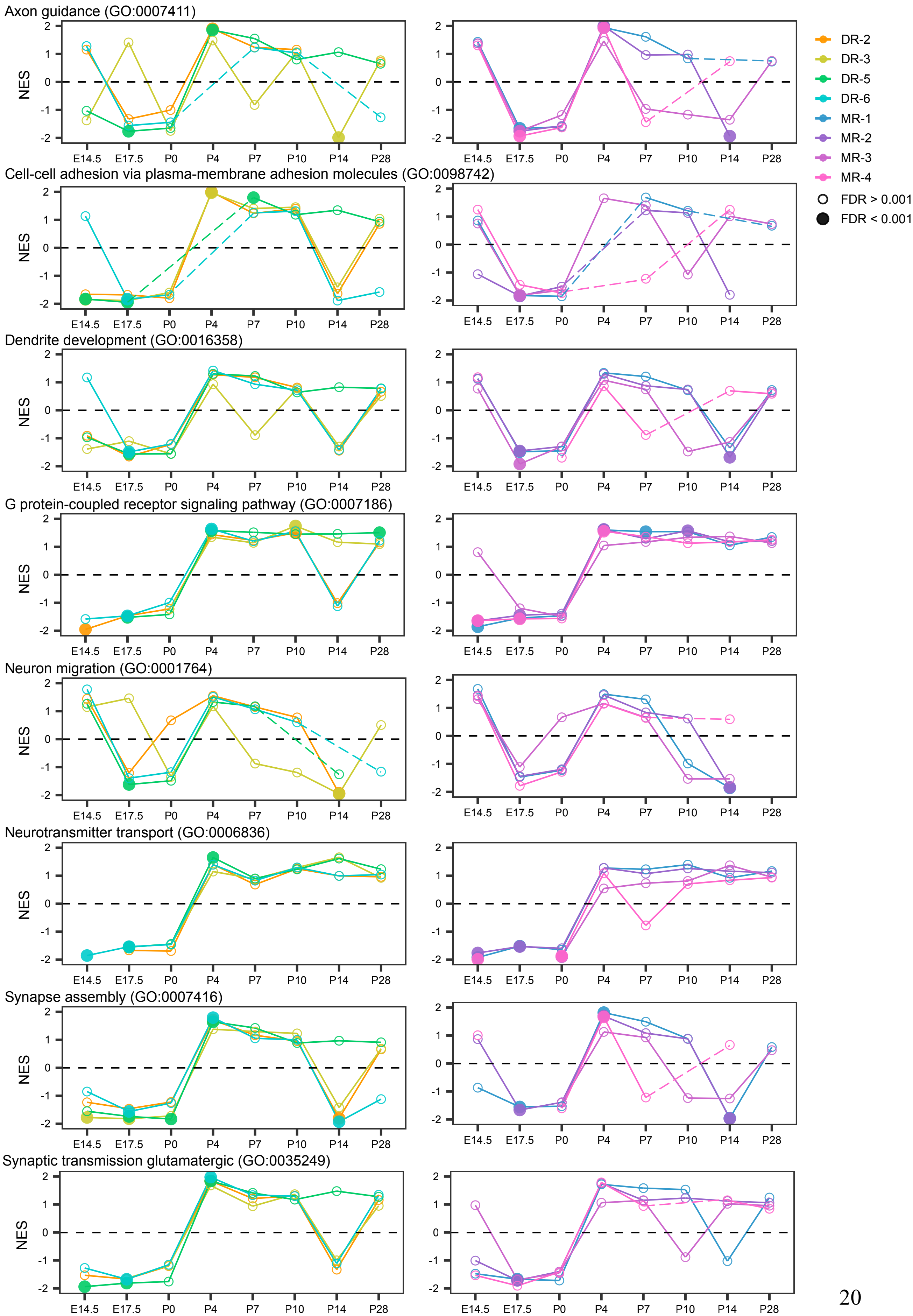

Axon guidance (GO:0007411)
Cell-cell adhesion via plasma-membrane adhesion molecules (GO:0098742)
Dendrite development (GO:0016358)
G protein-coupled receptor signaling pathway (GO:0007186)
Neuron migration (GO:0001764)
Neurotransmitter transport (GO:0006836)
Synapse assembly (GO:0007416)
Synaptic transmission glutamatergic (GO:0035249)
NES
E14.5
E17.5
P0
P4
P7
P10
P14
P28
DR-2
DR-3
DR-5
DR-6
MR-1
MR-2
MR-3
MR-4
FDR > 0.001
FDR < 0.001

Fig. S8. Enrichment of GO terms related to neuronal development in serotonin neuron subgroups across 8 developmental stages.

Normalized enrichment score (NES) of GO terms related to neuronal development for each subgroup of serotonin neurons across eight developmental stages. DR subgroups are shown on the left and MR subgroups are on the right. Filled circles indicate FDR <0.001. Missing circles are due to insufficient cells in some subgroup and developmental stage combinations for GSEA. Dashed lines indicate extrapolated trends across missing stages.

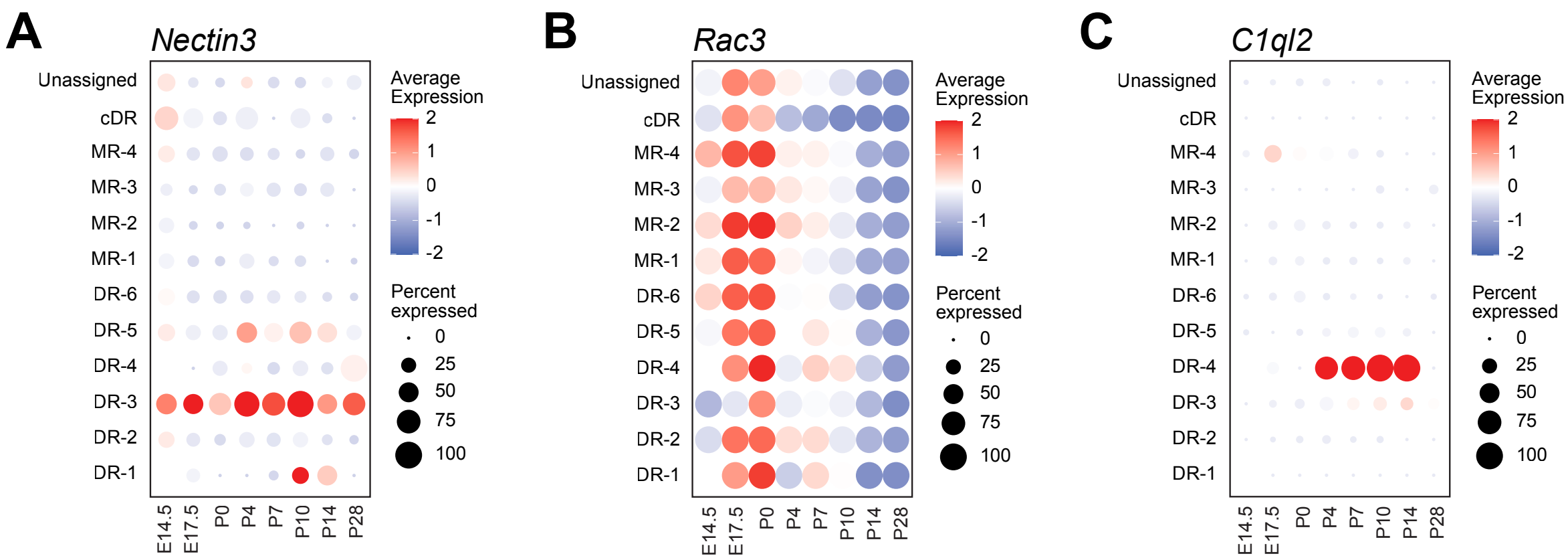

Fig. S9. Dotplots of representative subtype- and/or stage-specific genes related to axon guidance or synapse assembly.

A) Dotplot showing subtype-specific expression of Nectin3 across 8 developmental stages.

B) Dotplot showing E17.5 – P0 peaking pattern of Rac3 across all serotonin neuron subtypes.

C) Dotplot showing the subtype and stage specificity of C1ql2 expression.

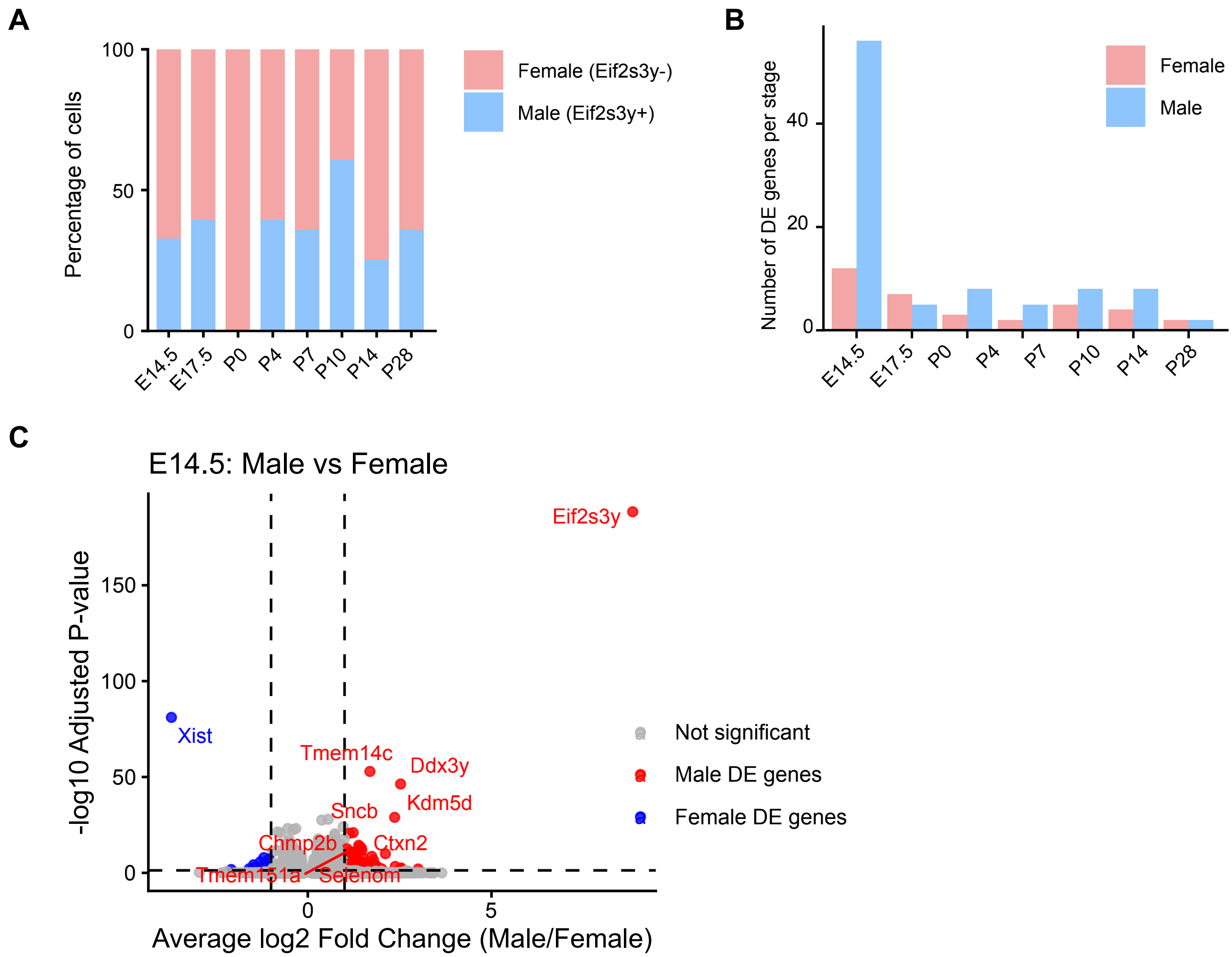

Fig. S10. Sex-associated transcriptional variation of serotonin neurons.

A) Percentage of serotonin neuron transcriptomes from each sex, determined by expression of Y-chromosome gene Eif2s3y, in each scRNA$^{DR\&MR}$ dataset across development.

B) Number of male- or female- specific differentially expressed (DE) genes in serotonin neurons at each developmental stage.

C) Volcano plot showing male- and female-specifc DE genes in E14.5 scRNA$^{DR\&MR}$ dataset. Top 10 DE genes are labelled.

Tables

Table S1. Table of average pseudotime values sorted by developmental stage and predicted cell body location in DR or MR.

STAGE	AVERAGE PSEUDOTIME PER STAGE (MEAN ± SD)	DR / MR	PSEUDOTIME FOR DR / MR (MEAN ± SD)
**E14.5**	2.18 ± 1.16	DR	1.53 ± 0.99
		MR	3.09 ± 0.63
**E17.5**	13.95 ± 3.58	DR	14.36 ± 4.04
		MR	13.40 ± 2.76
**P0**	14.67 ± 3.52	DR	16.51 ± 2.68
		MR	12.15 ± 2.92
**P4**	20.77 ± 4.33	DR	20.68 ± 3.39
		MR	20.86 ± 5.08
**P7**	23.37 ± 3.41	DR	22.30 ± 2.87
		MR	24.77 ± 3.55
**P10**	22.85 ± 3.32	DR	22.31 ± 2.77
		MR	23.43 ± 3.75
**P14**	28.43 ± 4.19	DR	28.45 ± 4.63
		MR	28.39 ± 2.78
**P28**	27.84 ± 2.72	DR	27.72 ± 2.75
		MR	27.99 ± 2.69

Table S2. Number of cells in each subgroup transferred from SmartSeq scRNA$^{DR\&MR}$ dataset across eight developmental stages

Stage	*cDR*	*DR-1*	*DR-2*	*DR-3*	*DR-4*	*DR-5*	*DR-6*	*MR-1*	*MR-2*	*MR-3*	*MR-4*	*Unassigned*
**E14.5**	4	0	53	35	0	73	105	85	90	14	36	435
**E17.5**	2	5	90	55	7	121	197	215	128	17	22	358
**P0**	10	6	97	37	8	123	116	148	82	53	49	318
**P4**	3	3	74	30	10	56	104	127	101	56	41	101
**P7**	9	25	52	84	13	66	55	71	66	90	72	81
**P10**	4	3	38	42	11	52	95	100	80	43	29	132
**P14**	12	8	146	73	7	43	80	61	35	48	14	47
**P28**	5	4	15	9	1	31	67	47	37	14	24	29

Movie S1 (separate file). Movie showing a 3D rendering of serotonin neuron axons in select cortical brain regions at developmental stage P4. The selected brain regions and their timestamps are as follows; ACA (0:00), PL (0:21), ORB (0:27), ILA (0:36), AI (0:43).

Movie S2 (separate file). Movie showing heatmaps of serotonin neuron axon densities across the whole mouse brain at P0, P4, P7 and P14 developmental stages. The heatmaps are depicted from a coronal perspective and the movie proceeds along the anterior-posterior axis.

Dataset S1 (separate file). Serotonin Neuron Axon Density and Statistical Analysis. Sheet 1: Average axon density values for 276 brain regions at P0, P4, P7, P10 and P14. Brain regions ordered based on the circular plot in Figure 1C Sheet 2. Statistics for the axon density changes between developmental stages based on ANOVA statistical analysis, Including a Dunnett Test column showing the stages with significant differences in axon density compared to P0.

Dataset S2 (separate file). Axon densities traced from individual *Gad2-Cre:Sert-Flp*, *Vglut3-Cre:Sert-Flp* nad *Piezo2-Cre:Sert-Flp* mouse brains harvested at P28.

Dataset S3 (separate file). Brain regions clustered by developmental serotonin innervation profiles. Only regions that existed in Dataset S1 and S2 were included for clustering (total: 186).

SI References

1. S. Migliarini, G. Pacini, B. Pelosi, G. Lunardi, M. Pasqualetti, Lack of brain serotonin affects postnatal development and serotonergic neuronal circuitry formation. *Mol Psychiatry* **18**, 1106–1118 (2013).

2. H. Ho, A. Fowle, M. Coetzee, I. H. Greger, J. F. Watson, An inhalation anaesthesia approach for neonatal mice allowing streamlined stereotactic injection in the brain. *Journal of Neuroscience Methods* **342**, 108824 (2020).

3. J. Chi, A. Crane, Z. Wu, P. Cohen, Adipo-Clear: A Tissue Clearing Method for Three-Dimensional Imaging of Adipose Tissue. *J Vis Exp* 58271 (2018). https://doi.org/10.3791/58271.

4. D. Friedmann, *et al.*, Mapping mesoscale axonal projections in the mouse brain using a 3D convolutional network. *Proc. Natl. Acad. Sci. U.S.A.* **117**, 11068–11075 (2020).

5. K. Van Den Berge, *et al.*, Trajectory-based differential expression analysis for single-cell sequencing data. *Nat Commun* **11**, 1201 (2020).

6. Z. Li, *et al.*, D-LMBmapX: Generalized Deep-Learning Pipeline for Automated Whole-brain Neural Circuitry Profiling Across Any Developmental Stages. [Preprint] (2025). Available at: http://biorxiv.org/lookup/doi/10.1101/2025.02.25.639766 [Accessed 26 February 2025].

7. Z. Li, *et al.*, D-LMBmap: a fully automated deep-learning pipeline for whole-brain profiling of neural circuitry. *Nat Methods* **20**, 1593–1604 (2023).

8. C. S. McGinnis, L. M. Murrow, Z. J. Gartner, DoubletFinder: Doublet Detection in Single-Cell RNA Sequencing Data Using Artificial Nearest Neighbors. *Cell Systems* **8**, 329-337.e4 (2019).

9. Y. Hao, *et al.*, Dictionary learning for integrative, multimodal and scalable single-cell analysis. *Nat Biotechnol* **42**, 293–304 (2024).

10. I. Korsunsky, *et al.*, Fast, sensitive and accurate integration of single-cell data with Harmony. *Nat Methods* **16**, 1289–1296 (2019).

11. F. Zhang, Y. Wu, W. Tian, A novel approach to remove the batch effect of single-cell data. *Cell Discov* **5**, 46 (2019).

12. B. Hie, B. Bryson, B. Berger, Efficient integration of heterogeneous single-cell transcriptomes using Scanorama. *Nat Biotechnol* **37**, 685–691 (2019).

13. K. Polański, *et al.*, BBKNN: fast batch alignment of single cell transcriptomes. *Bioinformatics* **36**, 964–965 (2020).

14. A. Gayoso, *et al.*, A Python library for probabilistic analysis of single-cell omics data. *Nat Biotechnol* **40**, 163–166 (2022).

15. N. Johansen, G. Quon, scAlign: a tool for alignment, integration, and rare cell identification from scRNA-seq data. *Genome Biol* **20**, 166 (2019).

16. K. Siletti, *et al.*, Transcriptomic diversity of cell types across the adult human brain. *Science* **382**, eadd7046 (2023).

17. K. Street, *et al.*, Slingshot: cell lineage and pseudotime inference for single-cell transcriptomics. *BMC Genomics* **19**, 477 (2018).

18. B. Van de Sande, *et al.*, A scalable SCENIC workflow for single-cell gene regulatory network analysis. *Nat Protoc* **15**, 2247–2276 (2020).

19. S. Xu, *et al.*, Using clusterProfiler to characterize multiomics data. *Nat Protoc* **19**, 3292–3320 (2024).

20. H. M. T. Choi, *et al.*, Third-generation in situ hybridization chain reaction: multiplexed, quantitative, sensitive, versatile, robust. *Development* **145**, dev165753 (2018).
